# Unified Control of a Powered Knee-Ankle Prosthesis Enables Walking, Stairs, Transitions, and Other Daily Ambulation Activities

**DOI:** 10.1109/TNSRE.2025.3595496

**Published:** 2025

**Authors:** Liam M. Sullivan, Marissa Cowan, Lukas Gabert, Tommaso Lenzi

**Affiliations:** Department of Mechanical Engineering and Robotics Center, The University of Utah, Salt Lake City, UT 84112 USA; Department of Mechanical Engineering and Robotics Center, The University of Utah, Salt Lake City, UT 84112 USA; Department of Mechanical Engineering and Robotics Center, The University of Utah, Salt Lake City, UT 84112 USA; Department of Mechanical Engineering, Robotics Center, the Rocky Mountain Center for Occupational and Environmental Health, and the Department of Biomedical Engineering, The University of Utah, Salt Lake City, UT 84112 USA.

**Keywords:** Biomechanics, control, lower-limb prosthesis, powered prosthetics, prosthetics

## Abstract

Conventional passive prostheses for lower limb amputees lack active assistance and cannot replicate the natural energy dynamics of healthy limbs, resulting in compromised mobility and diminished quality of life. Powered lower-limb prostheses can theoretically replicate the biomechanical function of healthy limbs during ambulation. However, existing powered prostheses fall short of fully restoring mobility for individuals with above-knee amputations. This limitation is mainly due to existing controllers, which struggle to coordinate their assistance with the user’s movements across a variety of activities in a natural manner. This paper proposes a novel unified controller for powered knee and ankle prostheses that enables multiple activities and adapts to users and the environment. The proposed controller enables natural ambulation without requiring explicit measurements of the environmental characteristics or classification of the intended ambulation task. In experiments with three amputee subjects, the proposed controller enabled walking on level, inclined, and uneven ground, ascending/descending stairs, sitting and standing, and seamless transitions between these activities. This work presents the first implementation of a unified, task-agnostic control strategy for continuous ambulation across everyday ambulation activities for powered knee and ankle prostheses.

## I. Introduction

CONVENTIONAL passive prostheses for lower-limb amputees do not provide active assistance during ambulation in the way that healthy limbs do [1]. As a result, ambulation with conventional passive lower-limb prostheses is slow, inefficient, and unstable [2], [3], resulting in limited mobility and diminished quality of life for amputees [4] Powered prostheses have the potential to address the limitations of conventional passive prostheses by closely matching the torque, speed, and power of biological legs during ambulation. Although many powered prostheses have been developed [5], [6], [7], [8], [9], [10], [11], [12], [13], [14], [15], available controllers still struggle to coordinate the movements of the prosthetic joints with the user and restore biomimetic mobility across a variety of tasks and conditions.

Most powered prosthesis controllers assume that ambulation is periodic and define the prosthesis’ movements based on the steady-state gait cycle of a specific ambulation task (e.g., walking, stair ascent) [16]. These controllers can then split the gait cycle into a few discrete, sequential phases [11], [17] or define a cycle as a continuous phase evolution [18], [19]. Position, impedance, or torque are then commanded based on the discrete or continuous gait phase.

Controllers that define discrete phases often use fixed impedance values for each phase, which is computationally inexpensive and can enable many ambulation activities [20], [21]. However, it requires manual tuning of the control parameters used within each state and the transition rules between states [22]. This manual tuning requires experts and can be time-consuming [23]. Additionally, each user must receive tuning specific to their gait, which limits the generalizability of this approach [23], [24].

Controllers that define a continuous phase evolution often enforce position, torque, or variable impedance trajectories derived from able-bodied datasets [18], [25], [26], [27]. This approach provides close-to-healthy biomechanics with minimal to no tuning, which may facilitate clinical translation. However, to enforce the appropriate biomechanics, the controller requires explicit knowledge of the environmental characteristics, such as walking speed [27] or ground incline [26]. This makes real-world implementation challenging. Additionally, by enforcing the average biomechanics of healthy, young subjects, this approach does not intrinsically account for user variability.

Regardless of the mid-level control approach, virtually all existing control methods require estimating the user’s intended ambulation task [28], [29], [30], [31]. The estimated task as well as transitions between tasks are determined using specific trigger conditions based, most commonly, on joint position or prosthesis loading [32], [33], [34]. Sensors in the prosthesis are then often integrated with supervised machine learning to determine the estimated task [35], [36], [37]. The accuracy of the classification depends on the amount of data used for training as well as the dimension of the classification space [38]. It can be improved using different kinds of sensors, including electromyography [39], [40], [41], sonomyography [42], range sensors [43], or cameras [44]. For the best-trained models, the rate of accurate classification is 98% [29], [45], [46], [47], [48]. However, users may take thousands of steps per day, and a single misclassified step can still lead to falls, injury, and users losing trust in the device [49]. Ambulation in the real world is unpredictable and requires continuous adaptation to the environment. Ambulation is often aperiodic, terrain conditions are continuously variable, and the desired task can change instantaneously. Therefore, using a machine learning classifier for ambulation in the real world may require nearly perfect online classification accuracy.

Existing powered prostheses have successfully replicated certain biological behaviors and enabled a variety of ambulation tasks under discrete, controlled conditions [16], [18], [26], [27], [50], [51], [52], [53], [54]. To adapt to variable inclines, previous controllers have successfully implemented shank-based ankle impedance as a bioinspired method of achieving intrinsic adaptation to the terrain [55]. To adapt to variable speeds, previous controllers have replicated minimum-jerk behavior from healthy biomechanics to adapt swing behavior and enable a smooth gait at any speed [56]. To enable sit-to-stand and stand-to-sit transitions, previous controllers have commanded biologically accurate torque profiles as well as virtual impedance to safely lift and lower users [50], [57]. To enable transitions between walking and stair ascent, previous controllers have manually switched between independent controllers specific to each activity [25]. While these previous approaches were successful in achieving normative ambulation for each specific task, none of these controllers incorporated all of these elements into a single controller.

To address the limitations in existing control strategies, this paper presents a controller for a powered knee and ankle prosthesis that builds upon the successful elements of previous controllers and incorporates them into a single, unified structure. The proposed controller enables walking at variable speeds and inclines and on rough terrains, ascending and descending stairs, sitting, and standing without enforcing able-bodied biomechanics or hand-tuning impedance parameters. Moreover, it enables all these activities and transitions between them without any task estimation. The proposed controller is furthermore intrinsically adaptable to the user and environment. We achieve this performance by combining a series of adaptive torque and impedance controllers that reproduce bioinspired behaviors rather than replicate normative biomechanics.

In this paper, we present the design of a unified controller and show the kinetics and kinematics of three amputee participants using the controller to perform various ambulation tasks both in the lab and in real-world settings. This paper demonstrates the generalizability of the proposed control strategy and validates its ability to enable biomimetic mobility. Furthermore, we demonstrate that the controller allows for real-world ambulation with a single amputee subject.

## II. Methods

### A. Unified Controller Structure

**Fig. 1.**
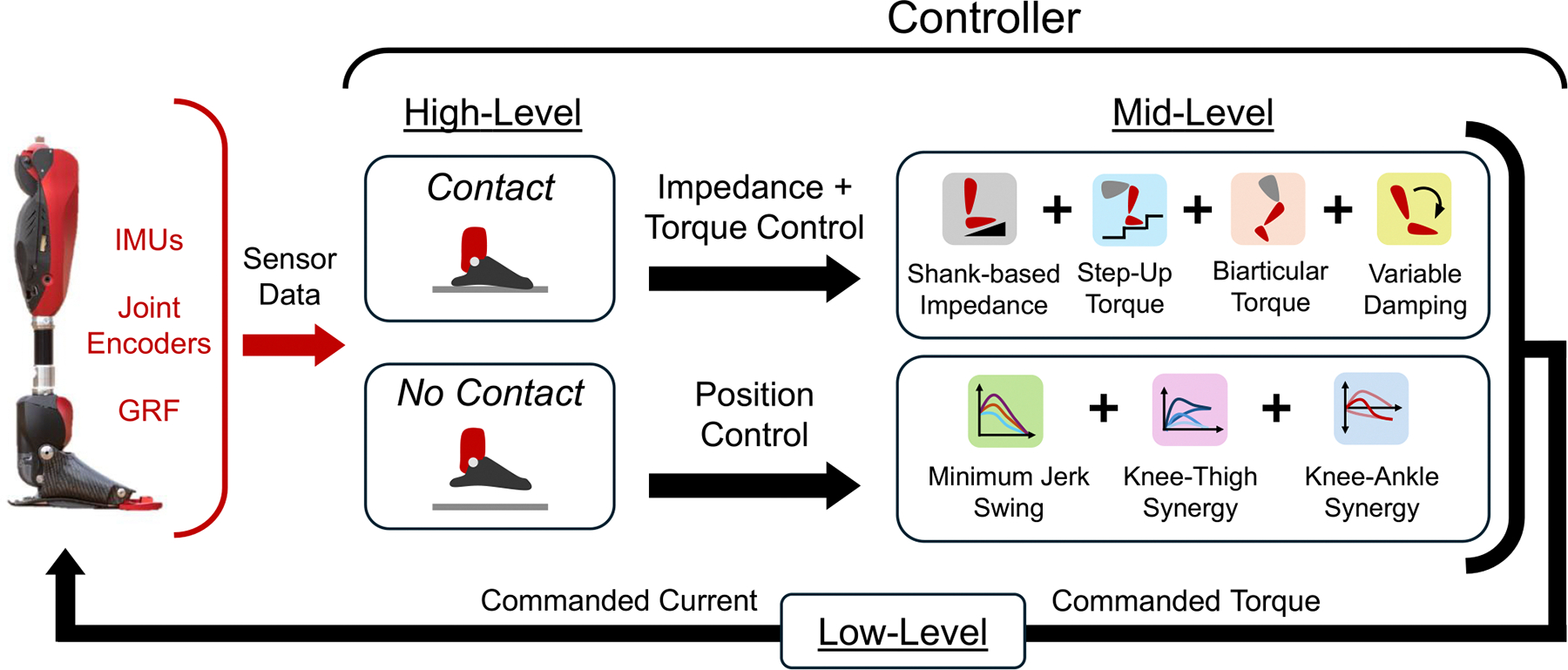
Control summary for the powered knee and ankle prosthesis, including sensor data, high-level state classification, mid-level torque calculation, and low-level current command.

A summary of the proposed controller is shown in Fig. 1. The proposed unified controller uses a simple finite-state machine with two states, *Contact* and *No Contact*, to indicate if the prosthetic foot is in contact with the ground. The finite-state machine switches from *Contact* when the vertical ground reaction force (GRF) is above 120 N to *No Contact* when the GRF is below 80 N. The transition from *Contact* to *No Contact* is called toe-off (TO), and the transition from *No Contact* to *Contact* is called heel-strike (HS), regardless of whether the prosthesis touches down with the heel or toe first. Specific knee and ankle controllers are used in each of the two states. The controller is robust to changes in the control state and avoids discontinuities when switching between them. As shown in the results later in this work, there were no discontinuities in control during transitions between *Contact* and *No Contact* (Fig. 11, Fig. S6, and Fig. S8).

#### 1) Knee Control During Contact:

In *Contact,* the total commanded torque, $T_{\mathrm{Knee}}$, is the sum of three components: Step-Up torque $\left(T_{\mathrm{Knee}}^{\mathrm{Step}-Up}\right)$, Biarticular torque ($T_{\mathrm{Knee}}^{\mathrm{Biart}}$), and Damping torque ($T_{\mathrm{Knee}}^{\mathrm{Damping}}$) (1).


(1) $$T_{\mathrm{Knee}}=T_{\mathrm{Knee}}^{\mathrm{Step}-Up}+T_{\mathrm{Knee}}^{\mathrm{Biart}}+T_{\mathrm{Knee}}^{\mathrm{Damping}}$$

**Fig. 2.**
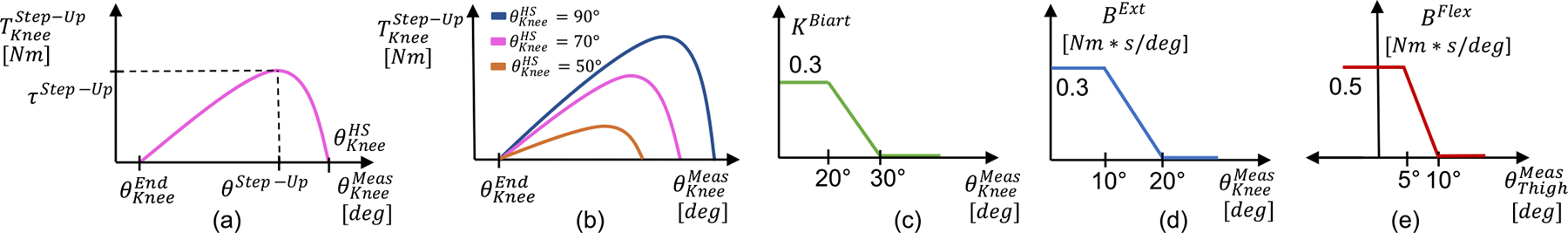
Step-Up torque knee control during *Contact*. (a) $T_{\mathrm{Knee}}^{\mathrm{Step}-Up}$ curve with maximum torque and angle of maximum torque defining the peak of the curve. (b) Sample step-up torque curves as a function of varying knee angle at HS, $\theta_{Knee}^{HS}$. damping coefficients. (c) Biarticular torque gain, $T_{\mathrm{Knee}}^{\mathrm{Biart}}$. (d) Extension, $B^{Ext}$, and (e) flexion, $B^{Flex}$, virtual knee damping coefficients.

The Step-up Torque, $T_{\mathrm{Knee}}^{\mathrm{Step-Up}}$, is intended to provide extension torque during stair ascent, sit-to-stand transitions, and similar movements (2). $T_{\mathrm{Knee}}^{\mathrm{Step-Up}}$ follows a bioinspired bell-shaped profile (Fig. 2a), which starts at 0 Nm at HS, when the measured knee prosthesis angle ($\theta_{\mathrm{Knee}}^{\mathrm{Meas}}$) is equal to the knee angle at heelstrike $\left(\theta_{\mathrm{Knee}}^{HS}\right)$. As the knee extends (i.e., $\theta_{\mathrm{Knee}}^{\mathrm{Meas}}$ decreases), $T_{\mathrm{Knee}}^{\mathrm{Step}-Up}$ increases until $\theta_{\mathrm{Knee}}^{\mathrm{Meas}}$ matches a specified peak-torque angle ($\theta^{\mathrm{Step-Up}}$), at which point the peak torque ($\tau^{\mathrm{Step}-Up}$) is achieved. $\theta^{\mathrm{Step}-Up}$ was tuned in this work for user preference. As the knee extends past $\theta^{\mathrm{Step}-Up}$, the torque decreases, finally reaching zero when $\theta_{\mathrm{Knee}}^{\mathrm{Meas}}$ equals the ending-torque angle $\left(\theta_{\mathrm{Knee}}^{\mathrm{End}}\right)$. $T_{\mathrm{Knee}}^{\mathrm{Step-Up}}$ is adapted online following (3)-(4). Specifically, the position at which the knee achieves the maximum torque ($\theta^{\mathrm{Step}-Up}$) increases proportionally to the difference between $\theta_{\mathrm{Knee}}^{HS}$ and $\theta_{\mathrm{Knee}}^{\mathrm{End}}$, where $\theta_{\mathrm{Knee}}^{\mathrm{End}}$ is set in these experiments to 5°. Moreover, $\tau^{\mathrm{Step}-Up}$ increases proportionally to the position of the knee at $\theta_{\mathrm{Knee}}^{HS}$ (4). As a result, as $\theta_{\mathrm{Knee}}^{HS}$ increases, higher extension torque is generated for the whole knee extension movement (Fig. 2b). Similar $T_{\mathrm{Knee}}^{\mathrm{Step}-Up}$ implementations were successfully used for stair ascent and sit-to-stand transitions in our prior studies [52], [57].


(2) $$T_{\mathrm{Knee}}^{\mathrm{Step}-Up}=f\left(\theta_{\mathrm{Knee}}^{HS},\theta^{\mathrm{Step}-Up},\tau^{\mathrm{Step}-Up}\right)$$

(3) $$\theta^{\mathrm{Step}-Up}=0.8*\left(\theta_{\mathrm{Knee}}^{HS}-\theta_{\mathrm{Knee}}^{\mathrm{End}}\right)+\theta_{\mathrm{Knee}}^{\mathrm{End}}$$

(4) $$\tau^{\mathrm{Step}-Up}=1.2\;*\;\theta_{\mathrm{Knee}}^{HS}$$

The Biarticular torque, $T_{\mathrm{Knee}}^{\mathrm{Biart}}$, is intended to replicate the function of the gastrocnemius muscle, which flexes the knee and plantarflexes the ankle in late stance. To this end, $T_{\mathrm{Knee}}^{\mathrm{Biart}}$ provides knee flexion torque proportionally to the commanded ankle torque ($T_{\mathrm{Ankle}}$), as defined by the biarticular gain ($K^{\mathrm{Biart}}$) following (5). Notably, $K^{\mathrm{Biart}}$ is not fixed but changes as a function of $\theta_{\mathrm{Knee}}^{\mathrm{Meas}}$ as shown in Fig. 2c, to prevent excessive knee flexion. Specifically, $K^{\mathrm{Biart}}$ is at a maximum value of 0.3 when $\theta_{\mathrm{Knee}}^{\mathrm{Meas}}<20^{\circ }$ and decreases linearly between $20^{\circ }<\theta_{\mathrm{Knee}}^{\mathrm{Meas}}<30^{\circ }$ to 0. Moreover, the maximum $T_{\mathrm{Knee}}^{\mathrm{Biart}}$ is capped at 5 Nm. This modulation mimics able-bodied behavior, where the muscle activation of the gastrocnemius decreases in late stance [58].


(5) $$T_{\mathrm{Knee}}^{\mathrm{Biart}}=min\left(\left(K^{\mathrm{Biart}}\cdot T_{\mathrm{Ankle}}\right),5\right)$$

The virtual damping torque, $T_{\mathrm{Knee}}^{\mathrm{Damping}}$, is intended to prevent the knee from buckling under the user’s weight, for example during standing and the early-stance phase of walking. The virtual damping works to smoothen the movements of the prosthetic knee during all ambulation activities. Damping coefficients are unique for extension and flexion. They are additionally modulated as a function of $\theta_{\mathrm{Knee}}^{\mathrm{Meas}}$ and $\theta_{\mathrm{Thigh}}^{\mathrm{Meas}}$ (Fig. 2d-e). Additional detail on $T_{\mathrm{Knee}}^{\mathrm{Damping}}$ is provided in supplementary material section S1a.

#### 2) Ankle Control During Contact:

During *Contact*, we define the total ankle torque, $T_{\mathrm{Ankle}}$, as the sum of virtual stiffness ($T_{\mathrm{Ankle}}^{\mathrm{Stiffess}}$) and virtual damping ($T_{\mathrm{Ankle}}^{\mathrm{Damping}}$) torques (6).


(6) $$T_{\mathrm{Ankle}}=T_{\mathrm{Ankle}}^{\mathrm{Stiffess}}+T_{\mathrm{Ankle}}^{\mathrm{Damping}}$$

**Fig. 3.**
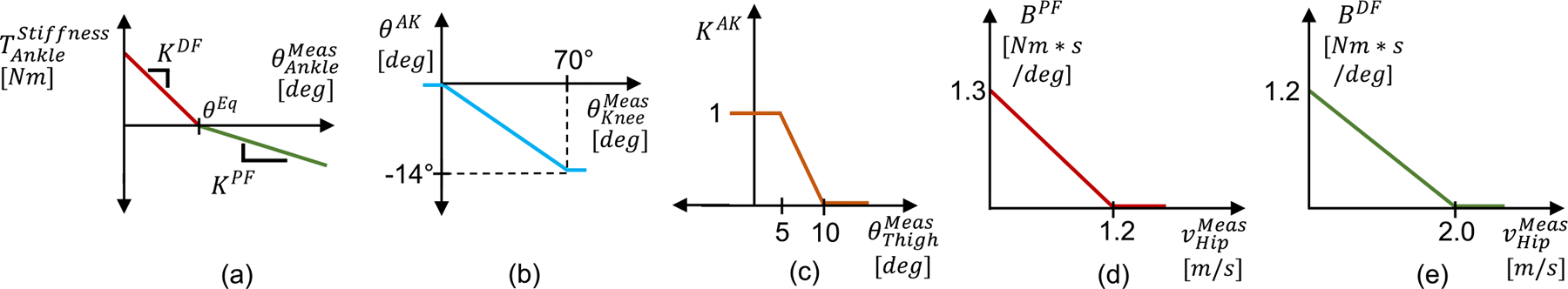
Ankle *Contact* control figures. (a) Virtual stiffness torque as a function of desired equilibrium angle and $K^{\mathrm{Stiffness}}$. (b) Knee-ankle synergy angle, $\theta^{AK}$. (c) Knee-ankle synergy gain, $K^{AK}$, as a function of thigh angle. (d) Plantarflexion, $B^{PF}$, and (e) dorsiflexion, $B^{DF}$, damping coefficients as functions of horizontal hip velocity, $v_{\mathrm{Hip}}^{\mathrm{Meas}}$.

The first element, $T_{\mathrm{Ankle}}^{\mathrm{Stiffness}}$, commands torque proportional to the deformation of a virtual spring, which is calculated as the difference between the current ankle angle ($\theta_{\mathrm{Ankle}}^{\mathrm{Meas}}$) and a desired equilibrium angle ($\theta^{Eq}$) (Fig. 3a). The deformation is then multiplied by a stiffness gain ($K^{\mathrm{Stiffness}}$) (7), which varies when the ankle angle is less than $\theta^{Eq}$ and greater than $\theta^{Eq}$ (8). $K^{Stiffness}$ was tuned in this work for user preference.

(7) $$T_{\mathrm{Ankle}}^{\mathrm{Stiffness}}=K^{\mathrm{Stiffness}}\;*\;\left(\theta_{\mathrm{Ankle}}^{\mathrm{Meas}}-\theta^{Eq}\right)$$

(8) $$K^{\mathrm{Stiffness}}=\left\{\begin{array}{ll} K^{DF}=4, & \mathrm{if}\;\theta_{\mathrm{Ankle}}^{\mathrm{Meas}}<\theta^{Eq}\\ K^{PF}=1, & \mathrm{if}\;\theta_{\mathrm{Ankle}}^{\mathrm{Meas}}\geq \theta^{Eq} \end{array}\right.$$

$\theta^{Eq}$ is the sum of two terms, $\theta^{Eq1}$ and $\theta^{Eq2}$ (9).


(9) $$\theta^{Eq}=\theta^{Eq1}+\theta^{Eq2}$$

(10) $$\theta^{Eq1}=min\left(\left(\theta_{\mathrm{Ankle}}^{\mathrm{Meas}}+\theta_{\mathrm{Shank}}^{\mathrm{Meas}}\right),\pm 8\right)$$

(11) $$\theta^{Eq2}=K^{AK}\;*\;\theta^{AK}$$

$\theta^{Eq1}$ is intended to enable the ankle prosthesis to adapt to different slopes and uneven terrain. $\theta^{Eq1}$ is defined as the sum of $\theta_{\mathrm{Ankle}}^{\mathrm{Meas}}$ and the prosthesis’s shank angle with respect to gravity ($\theta_{\mathrm{Shank}}^{\mathrm{Meas}}$) and is limited to a maximum of 8° in either plantarflexion or dorsiflexion (10). $\theta^{Eq1}$ creates a virtual spring acting on the shank orientation with respect to the vertical gravity vector. Creating a virtual spring around the shank orientation allows the prosthetic ankle to produce biomechanically accurate torque/angle profiles during ambulation on slopes and uneven terrain without explicitly estimating the slope [55]. Notably, $\theta^{Eq1}$ is constrained to 0° when $\theta_{\mathrm{Shank}}^{\mathrm{Meas}}$ is less than −5°. This is intended to create consistent and stable ankle behavior in early stance.

$\theta^{Eq2}$ is intended to enable the ankle prosthesis to dorsiflex when the knee flexes during activities like stand-to-sit transitions and stair climbing. To this end, $\theta^{Eq2}$ increases proportionally to the ankle-knee synergy angle ($\theta^{AK}$) based on the ankle-knee synergy gain ($K^{AK}$) (11) (Fig. 3b). We use the descriptor “synergy” to define variables that linearly modify either the knee or ankle based on the behavior of another segment of the prosthesis or body. $K^{AK}$ is not fixed but changes with the thigh angle (Fig. 3c). Specifically, $K^{AK}$ is equal to 1 when $\theta_{\mathrm{Thigh}}^{\mathrm{Meas}}<5^{\circ }$ and decreases to 0 linearly between $5^{\circ }<\theta_{\mathrm{Thigh}}^{\mathrm{Meas}}<10^{\circ }$. Because of this dependency of $K^{AK}$ to the thigh angle, $\theta^{Eq2}$ is different from zero only when the thigh is in front of the user, as is the case, for example, during sitting or squatting. On the other hand, $\theta^{Eq2}$ is zero when the thigh is behind the user, which prevents the ankle from dorsiflexing during late stance when ankle plantarflexion is needed for pushoff [59].

$T_{\mathrm{Ankle}}^{\mathrm{Damping}}$ is commanded dynamically to allow for biomimetic energy injection. Specifically, the controller varies damping as a function of hip velocity, $v_{Hip}^{\mathrm{Meas}}$, to provide less damping at higher hip velocity and thus allow more positive energy from the ankle stiffness (Fig. 3d, e). Additional detail on $T_{\mathrm{Ankle}}^{\mathrm{Damping}}$ can be found in supplementary material section S1b.

#### 3) Knee Control During No Contact:

During *No Contact*, we calculate a desired knee position and utilize a PID controller with loose gains. $\theta_{\mathrm{Knee}}^{\mathrm{Des}}$ is the sum of the minimum-jerk angle ($\theta_{\mathrm{Knee}}^{MJ}$) and the thigh-to-knee synergy angle ($\theta_{\mathrm{Knee}}^{\mathrm{Syn}}$). A synergy gain ($K^{\mathrm{Syn}}$) amplifies $\theta_{\mathrm{Knee}}^{\mathrm{Syn}}$ and attenuates $\theta_{\mathrm{Knee}}^{MJ}$ as it varies from 0 to 1 (12).


(12) $$\theta_{\mathrm{Knee}}^{\mathrm{Des}}=\theta_{\mathrm{Knee}}^{MJ}*\left(1-K^{\mathrm{Syn}}\right)+\theta_{\mathrm{Knee}}^{\mathrm{Syn}}\cdot K^{\mathrm{Syn}}$$

$\theta_{\mathrm{Knee}}^{MJ}$ aims to move the knee joint from its angle at TO to a fully extended angle in preparation for HS while mathematically maximizing smoothness. This approach is inspired by the minimum-jerk behavior in healthy biomechanics and is derived from a previous implementation in a powered prosthesis controller [56]. $\theta_{\mathrm{Knee}}^{MJ}$ changes as a function of the knee angle at TO ($\theta_{\mathrm{Knee}}^{TO}$), the knee velocity at TO (${\dot{\theta }}_{\mathrm{Knee}}^{TO}$) and the desired trajectory duration ($t^{\mathrm{swing}}$) (13), as shown in Fig. a-b.


(13) $$\theta_{\mathrm{Knee}}^{MJ}=f\left(\theta_{\mathrm{Knee}}^{TO},{\dot{\theta }}_{\mathrm{Knee}}^{TO},t^{\mathrm{swing}}\right)$$

**Fig. 4.**
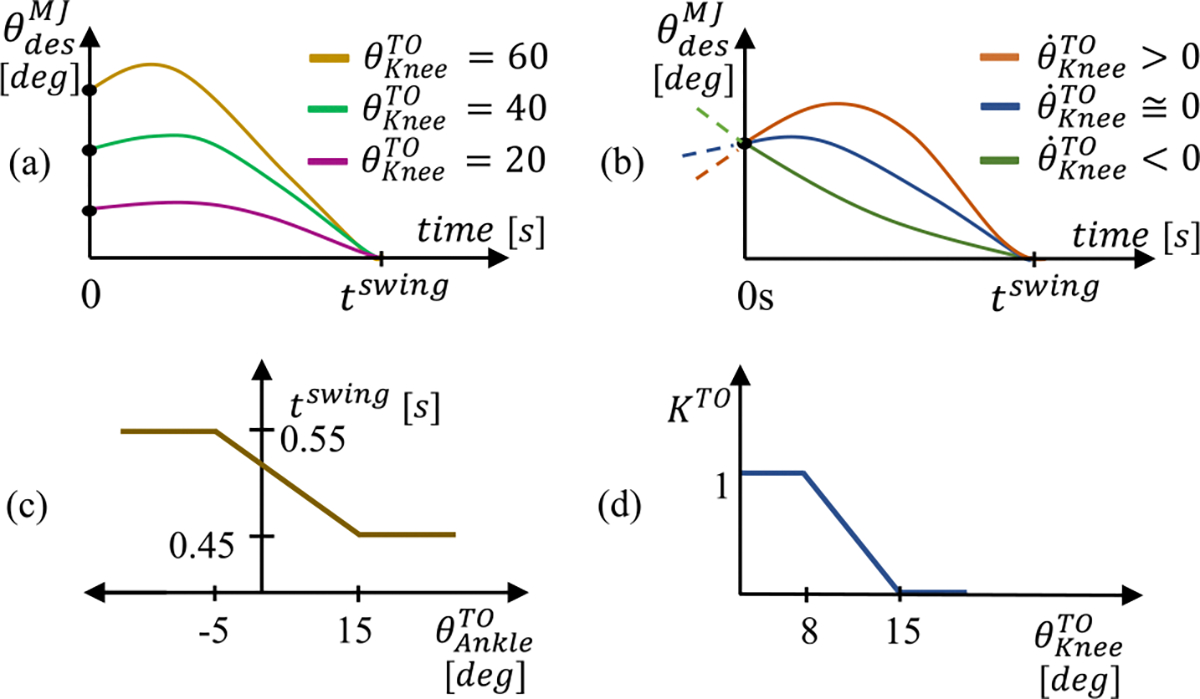
Minimum Jerk Trajectory control for *No Contact*. (a) Minimum-jerk swing trajectories for sample knee angles at TO and (b) swing trajectories for positive, zero, and negative knee velocities at TO. (c) Knee swing trajectory time, $t^{\mathrm{swing}}$, as a function of ankle angle at TO. (d) TO term, $K^{TO}$, calculated as a component of $K^{Syn}$.

$t^{\mathrm{swing}}$ changes as a function of ankle angle at TO $\left(\theta_{\mathrm{Ankle}}^{TO}\right)$ as shown in Fig. 4c. Specifically, $t^{\mathrm{swing}}$ is at a maximum value of 0.55s when $\theta_{\mathrm{Ankle}}^{TO}<-5^{\circ }$ and decreases linearly to 0.45s when $\theta_{\mathrm{Ankle}}^{TO}>15^{\circ }$. Thus, the greater the ankle plantarflexion angle at TO, the shorter the knee swing movement. This behavior is inspired by nonamputee biomechanics, which shows that as walking speed increases, swing duration decreases [60], [61]. $t^{\mathrm{swing}}$ was tuned based on user preference.

The thigh-to-knee synergy angle, $\theta_{\mathrm{Knee}}^{\mathrm{Syn}}$, is intended to coordinate the movements of the prosthetic knee with that of the residual limb (i.e., the user’s thigh on the amputation side) as needed for activities like stair climbing. As the user flexes their residual thigh, $\theta_{\mathrm{Knee}}^{\mathrm{Syn}}$ commands biomimetic knee flexion. This approach was previously presented in [52]. The detailed calculation of $\theta_{\mathrm{Knee}}^{\mathrm{Syn}}$ is described in supplementary material section S2a.

As shown in (12), $\theta_{\mathrm{Knee}}^{MJ}$ and $\theta_{\mathrm{Knee}}^{\mathrm{Syn}}$ are combined to obtain $\theta_{\mathrm{Knee}}^{\mathrm{Des}}$ based on the synergy gain $K^{\mathrm{Syn}}$, which is the sum of constant value calculated at TO ($K^{TO}$), and a delta value (ΔK), which is updated continuously (14).


(14) $$K^{Syn}=max\left(min\left(\left(K^{TO}+\Delta K\right),1\right),0\right)$$

$K^{TO}$ is calculated at TO as a function of $\theta_{\mathrm{Knee}}^{TO}$ as shown in Fig. 4d. Specifically, $K^{TO}$ is at a maximum value of 1 when $\theta_{\mathrm{Knee}}^{TO}<8^{\circ }$ and decreases linearly to 0 between 8° and 15°. Thus, when the knee angle at TO is less than 8°, $K^{TO}$ is 1 and $\theta_{\mathrm{Knee}}^{\mathrm{Des}}$ at TO is equal to $\theta_{\mathrm{Knee}}^{\mathrm{Syn}}$. In contrast, when the knee angle at TO is above 15°, $K^{TO}$ is 0 and $\theta_{\mathrm{Knee}}^{\mathrm{Des}}$ at TO is equal to $\theta_{\mathrm{Knee}}^{MJ}$. The ΔK term can modify $K^{\mathrm{Syn}}$ after the initial value is set at TO. If $\theta_{\mathrm{Thigh}}^{\mathrm{Meas}}$ and ${\dot{\theta }}_{\mathrm{Thigh}}^{\mathrm{Meas}}$ are both low, ΔK is set to a negative value so that $K^{\mathrm{Syn}}$ decreases to 0 over time. This decrease in $K^{\mathrm{Syn}}$ means $\theta_{\mathrm{Knee}}^{\mathrm{Des}}$ follows $\theta_{\mathrm{Knee}}^{MJ}$ which is common for walking activities (15). If the user flexes their thigh beyond a threshold angle and ${\dot{\theta }}_{\mathrm{Thigh}}^{\mathrm{Meas}}$ is high, ΔK is set to a positive value, which results in $K^{\mathrm{Syn}}$ increasing to 1. This increase in $K^{\mathrm{Syn}}$ means $\theta_{\mathrm{Knee}}^{\mathrm{Des}}$ follows $\theta_{\mathrm{Knee}}^{\mathrm{Syn}}$, which is common for activities like stair ascent. ΔK is updated every control-loop iteration (i.e., every 2 ms). Also, $K^{\mathrm{Syn}\;}$ cannot go above 1 or below 0.


(15) $$\text{Δ}K=\left\{\begin{array}{ll} 0.016, & \;\text{if}\;\theta_{\mathrm{Thigh}\;}^{\mathrm{Meas}\;}<-35\\ & \;\text{and}\;{\dot{\theta }}_{\mathrm{Thigh}\;}^{\text{Meas}\;}<-20\\ & \;\text{and}\;\theta_{\mathrm{Knee}\;}^{\mathrm{Meas}\;}>20\\ -0.008, & \;\text{if}\;-10>\theta_{\mathrm{Thigh}\;}^{\mathrm{Meas}\;}\\ & \;\text{and}\;\theta_{\mathrm{Thigh}\;}^{\mathrm{Meas}\;}>-35\\ & \;\text{and}\;{\dot{\theta }}_{\mathrm{Thigh}\;}^{\text{Meas}\;}>-50 \end{array}\right.$$

#### 4) Ankle Control During No Contact:

During *No Contact*, we calculate the desired ankle position ($\theta_{\mathrm{Ankle}}^{\mathrm{Des}}$) and enforce it using a loosely tuned PID controller. $\theta_{\mathrm{Ankle}}^{\mathrm{Des}}$ is the product of the synergy coefficient, $K^{\mathrm{Syn}}$ (14) and a synergistic ankle angle $\left(\theta_{\mathrm{Ankle}}^{\mathrm{Syn}}\right)$ (16).


(16) $$\theta_{\mathrm{Ankle}}^{\mathrm{Des}}=\theta_{\mathrm{Ankle}}^{\mathrm{Syn}}\cdot K^{\mathrm{Syn}}$$

$\theta_{\mathrm{Ankle}}^{\mathrm{Syn}}$ dorsiflexes the ankle as a function of knee flexion and residual thigh acceleration. The detailed calculation of $\theta_{\mathrm{Ankle}}^{\mathrm{Syn}}$ is described in supplementary material section S2b.

### B. Hardware

**Fig. 5.**
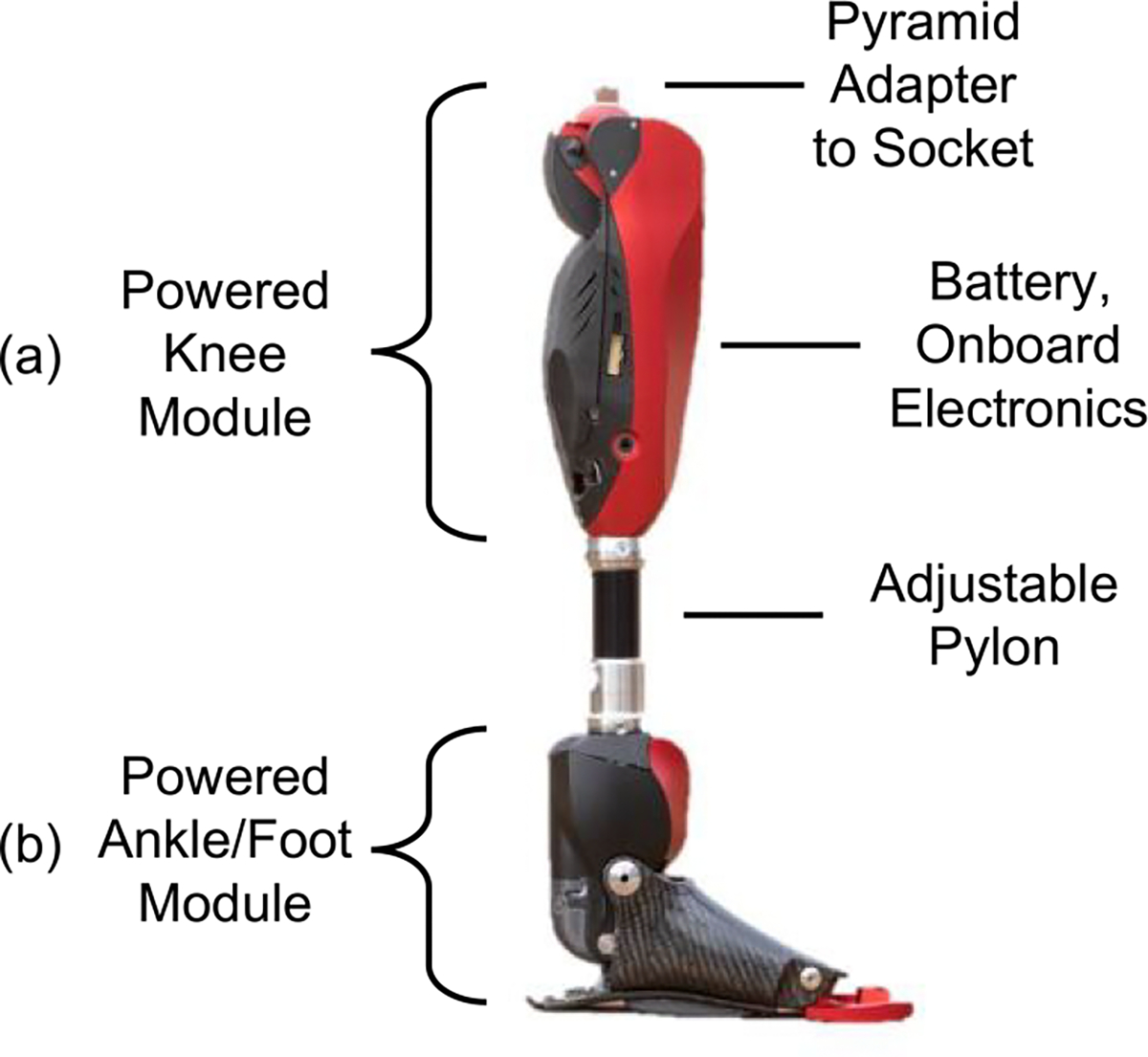
The Utah Bionic leg used in all experiments. Independent (a) knee and (b) ankle modules.

We tested the proposed controller with the Utah Bionic Leg, a powered knee and ankle prosthesis previously developed in the HGN Lab for Bionic Engineering (Fig. 5) [62]. The Utah Bionic leg is a self-contained, battery-powered, lightweight robotic prosthesis with knee and ankle/foot modules (Fig. 5). The powered knee module uses a torque-sensitive actuator that combines the benefits of variable transmissions and series elastic actuators [63]. The powered ankle/foot module uses an underactuated series elastic actuator, which actuates the ankle and toe joint and allows for energy regeneration [5]. Each joint can produce up to 150 Nm of torque [62]. The knee and ankle have active ranges of motion of 120° and 40°, respectively [62]. A custom instrumented pyramid adapter is attached to the top of the ankle module to estimate the vertical ground reaction force (GRF) and sagittal plane torques [64], which are necessary for the proposed unified controller. The prosthesis houses three IMUs placed in the foot and shank, as well as on the top of the knee to measure the thigh. Joint encoders at the knee and ankle measure angular position. The onboard electronics run the proposed control algorithm at 500 Hz and communicate via Wi-Fi to a remote computer for data telemetry and parameter tuning; however, the device can run without remote connection. The knee and ankle/foot modules are connected by a standard 30-mm diameter pylon, which is cut to the appropriate length to accommodate different user heights and residual limb lengths.

### C. Experimental Protocol

For this study, we recruited three subjects with unilateral above-knee amputation and mobility level K3 based on Medicare Functional Classification Level [65]. The number of subjects in this study was selected to demonstrate the functionality of the proposed controller. These subjects provide variability in body mass and height to achieve this goal (Table I). The Institutional Review Board at the University of Utah approved the study protocol (IRB #00103197). The subjects provided informed consent to participate in the study and for the publication of photos and videos from the experiment. Subject information can be found in Table I. A certified prosthetist fit and aligned the prosthesis before all experiments. Each subject performed the activities in this paper during a 3-hour testing period. Through participation in prior studies, each subject had more than 10 hours of experience with different iterations of the prosthesis and control algorithm. During the 3-hour testing period, each subject practiced using the prosthesis for up to 30 minutes and received verbal training from the experimenters. During the same period, the experimenters spent 15 minutes tuning up to eight of the control parameters for user preference, if desired, including: $\theta^{\mathrm{Step-Up}}$ (2), $K^{PF}$ (8), $K^{DF}$ (8), $t^{\mathrm{swing}}$ (13), $B^{\mathrm{Ext}}$ (S2), $B^{\mathrm{Flex}}$ (S2), $B^{PF}$ (S4), and $B^{DF}$ (S4).

**Fig. 6.**
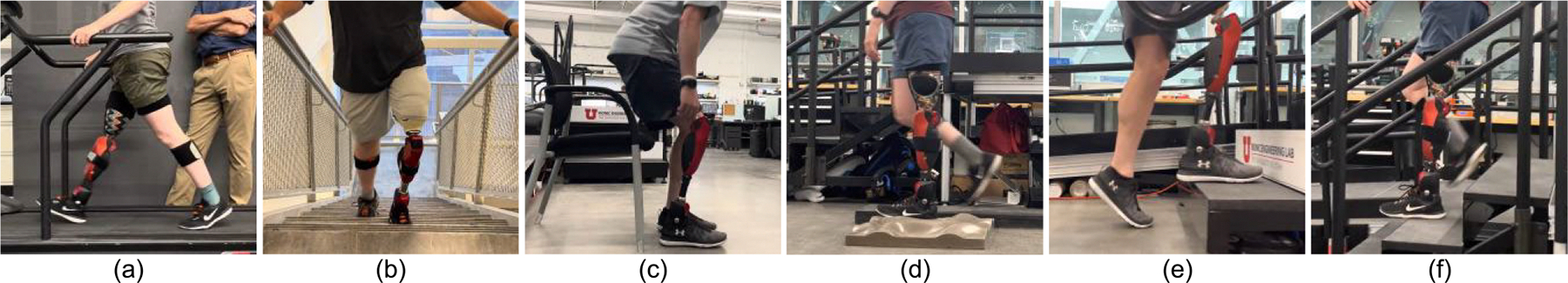
Subjects performing the experiments described in the text: (a) level ground walking, (b) stair ascent, (c), sit-to-stand, (d) walking on uneven terrain, (e) walk to stair ascent transition, and (f) walk to stair descent transition.

The subjects then performed a series of tests to validate the performance of the proposed controller. These tests included (1) walking on a treadmill at 1.0 m/s and inclines of 0%, 5%, and 10% for 60 seconds each after reaching steady state speed, (2) walking on a level treadmill at 0.72 m/s, 1 m/s, and 1.25 m/s for 60 seconds each after reaching steady state speed, (3) 10 trials of taking two consecutive strides on Sensa semi-rigid terrain patches (Ottobock, Item #: 752T1) and then two strides on level ground, (4) climbing a flight of 10 stairs three times, (5) sitting and standing from a chair continuously during a 60 second period, and (6) five trials of transition sequences from walking on level ground to climbing stairs and then transitioning back to walking, performing both stair ascent and descent variations and leading with both their prosthesis and sound side first for each transition, resulting in eight total transitions. Finally, a single subject performed a real-world ambulation demonstration which included all previously tested activities and more in a continuous outdoor sequence. Fig. 6 shows images of subjects performing the laboratory experiments. A video of each experiment being performed by subjects can be found in supplementary materials. There are additional results and figures presented in supplementary materials which analyze the same experiments. Those figures show the contributions of individual control elements to provide detailed understanding of the intent and performance of the proposed controller.

### D. Data Processing

Joint kinematics and GRF data were recorded at 500 Hz by sensors embedded in the prosthesis. Data was processed offline in MATLAB. When analyzing individual strides, we first segmented each stride from prosthesis heel strike to prosthesis heel strike, using the GRF readings. Then we interpolated each stride to 1000 samples and computed average joint trajectories for three trials of that stride. When analyzing a series of strides, we segmented the strides from each trial individually. We then averaged the data from three of the same stride type from different trials of the experiment and then sequenced the averaged strides in order. This provides interpolated, averaged data from three trials of each experiment.

## III. Results

### Steady State Activities

#### A. Walking at Variable Inclines and Speeds

**Fig. 7.**
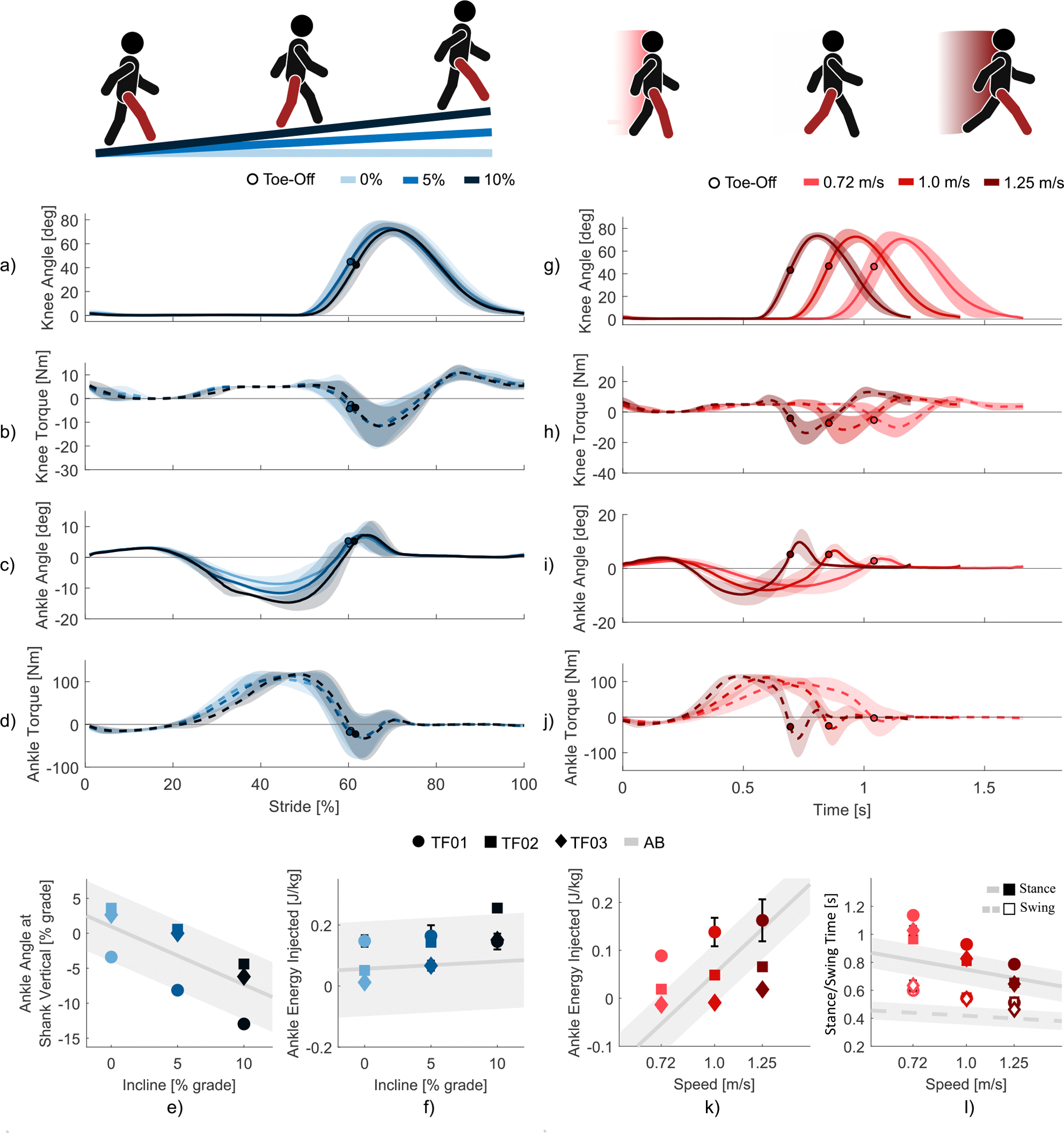
Variable Incline and Variable Speed walking. (a) Knee angle, (b) knee torque, (c) ankle angle, and (d) ankle torque from HS to HS at 0%, 5%, and 10% inclines. (e) Ankle angle when the shank angle is 0° during midstance of walking. (f) Energy injected by the ankle during the stance phase of walking at variable inclines. (g) Knee angle, (h) knee torque, (i) ankle angle, and (j) ankle torque from HS to HS at 0.72 m/s, 1.0 m/s, and 1.25 m/s. (k) Energy injected by the ankle during the stance phase of walking at variable speeds. (l) Stance and swing times at variable speeds. All subject data in (a-d) and (g-j) represent 30 strides total, 10 from each subject, with a solid line representing average values and the shaded region representing ± one standard deviation. In (e, f) and (k, l), unique symbols represent the average of 10 strides from each subject, with error bars representing ± one standard deviation. All able-bodied reference data is from Carmago et al. and represents averaged values from 30 strides across five subjects, shown as a solid or dashed line, and shaded regions represent ± one standard deviation [66].

All three amputee users successfully walked on variable inclines as the prosthesis biomimetically adapted ankle behavior. Fig. 7a-f shows joint data from three amputee subjects walking on a treadmill at 1.0 m/s at inclines of 0%, 5%, and 10% grade. HS and TO events are determined by the GRF sensor described in IIA. HS and TO events from AB data are determined by motion capture. The subjects’ knee trajectory did not vary substantially across the three inclines. The average peak knee angle varied between 72.4 +/− 6.3° and 74.8 +/− 5.2° and TO occurred between 59% and 61% of the stride (Fig. 7a). Peak knee angles from able-bodied biomechanics across similar speeds range from 64.6° to 70.8° between 75% and 76% of stride [66]. More considerable adaptation occurred in the ankle, with a noticeable trend in dorsiflexion as ramp incline increased. The average peak ankle dorsiflexion angle decreased from −8.9 +/−2.4° to −12.0 +/− 3.1° to −15.0 +/− 2.4° at 0%, 5%, and 10%, respectively (Fig. 7c). Peak ankle angles from able-bodied biomechanics across similar speeds range from −4.2° to −8.6° [66]. We measured ankle angle at the point that the shank is vertical with respect to gravity, which occurs during midstance, to assess the ankle’s adaptation to incline (Fig. 7e). With the foot flat on the treadmill at this point, ankle angle decreased proportionally as ramp incline increased. Converting ankle angle to % grade for comparison, average ankle grade was 0.9%, −2.5%, and −7.9% at 0%, 5%, and 10% grade inclines, respectively (Fig. 7e). This result reasonably matches able-bodied data, in which average ankle grade is 0.9%, −3.2%, and −7.3% at the three tested inclines (Fig. 7e). As incline increased, ankle adaptation during stance resulted in greater ankle torque during pushoff and therefore higher positive energy injection (Fig. 7f). Average energy injected by the ankle, normalized by individual weight, was 0.07 J/kg, 0.12 K/kg, and 0.18 J/kg at 0%, 5%, and 10% grade inclines, respectively. This result follows the same trend in able-bodied data, in which average energy injected is 0.06 J/kg, 0.07 J/kg, and 0.08 J/kg at the three tested inclines (Fig. 7f). The three amputee users successfully walked on three different inclines as the prosthesis provided a natural increase in ankle dorsiflexion and higher positive energy injection as incline increased.

All three amputee users were able to walk at variable speeds as the prosthesis biomimetically adapted knee and ankle behavior. Fig. 7g-l shows joint data from three amputee subjects walking on a level treadmill at speeds of 0.72 m/s, 1.0 m/s, and 1.25 m/s. Peak knee angle and TO point did not vary substantially across the three inclines, with peak knee angles ranging from 73.2 +/− 3.7° to 74.1 +/− 4.1° and TO occurring between 58% and 62% of the stride (Fig. 7g). Peak knee angles from able-bodied biomechanics across similar speeds range from 64.6° to 67.0° between 75% and 76% of stride [66]. In the ankle, however, walking at faster speeds resulted in a significant increase in both dorsiflexion during midstance and plantarflexion at TO. The average peak ankle dorsiflexion angle decreased from −6.8 +/− 2.9° to −8.1 +/− 2.8° to −9.8 +/− 4.0° at 0.72 m/s, 1.0 m/s, and 1.25 m/s, respectively (Fig. 7i). Peak ankle dorsiflexion angles from able-bodied biomechanics across similar speeds range from −13.1° to −15.7° [67]. The average ankle plantarflexion angle at TO increased from 3.4 +/− 1.8° to 5.5 +/− 2.6° to 7.1 +/− 2.8° at the three tested speeds (Fig. 7i). Peak ankle plantarflexion angles from able-bodied biomechanics across similar speeds range from 13.8° to 16.4° [66]. Increasing plantarflexion angle is a result of the ankle providing higher pushoff torque when walking at faster speeds. Higher pushoff torque increases the total energy injected by the ankle. At 0.72 m/s, 1.0 m/s, and 1.25 m/s the average ankle energy injected across the three subjects was 0.03 J/kg, 0.06 J/kg, and 0.08 J/kg, respectively (Fig. 7k). This result follows the same trend in able-bodied data, in which average energy injected is −0.05 J/kg, 0.05 J/kg, and 0.14 J/kg at the three tested speeds [67]. The users experienced adaptation to walking speed during both stance and swing. As speed increased across the three tests, the average total stride time decreased from 1.66 s to 1.40 s to 1.20 s (Fig. 7l). Stride time from able-bodied biomechanics across similar speeds ranges from 1.2s to 1.0s [66]. During stance, ankle adaptation allowed the user to rollover the foot faster, resulting in decreasing stance times of 1.04 s, 0.85 s, and 0.70 s (Fig. 7l). During swing, the duration of the swing trajectory decreased as ankle plantarflexion increased, resulting in decreasing swing times of 0.61 s, 0.54 s, and 0.49 s (Fig. 7l). The three users successfully walked at three variable speeds as the prosthesis allowed greater range of motion in the ankle, injected higher positive energy, and decreased swing duration as speed increase.

#### B. Rough Terrain

**Fig. 8.**
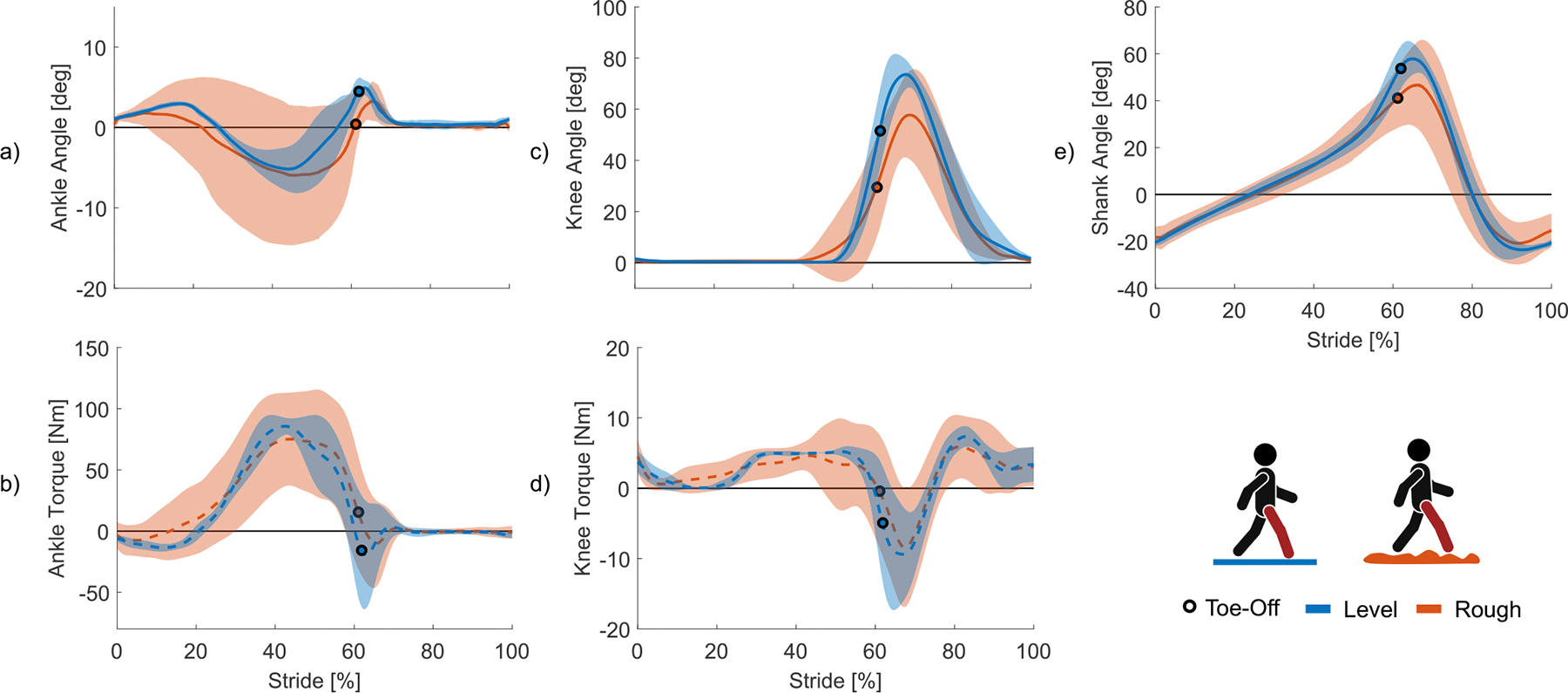
Variable Terrain walking. (a) Ankle angle, (b) ankle torque, (c) knee angle, (d) knee torque, and (e) shank angle from HS to HS walking at a self-selected speed on level ground and variable, uneven terrain. All data represents 30 strides total, 10 from each subject. All solid lines represent mean values and shaded regions represent ± one standard deviation.

All three amputee users were able to walk on variable, “rough” terrain as the prosthesis adapted ankle behavior. Fig. 8 shows ankle, shank, and knee positions as well as ankle and knee torque from three amputee subjects walking on level ground and rough terrain. The rough terrain consisted of randomly dispersed hills and valleys, varying in height from 20–80mm at inclines and declines of 10–40° (Ottobock, Item #: 752T1). HS and TO events are determined by the GRF sensor described in IIA. Ankle behavior varied substantially when walking on rough terrain (Fig. 8a). This variation is particularly evident in the increased range of motion of the ankle on rough terrain compared to level ground. The knee and shank, in contrast, have more consistent behavior between the two conditions (Fig. 8c, e). To assess the prosthesis’ adaptability as the user steps on highly inclined and declined terrain, we measure the standard deviation of the ankle, shank, and knee positions during stance. On level ground, the average standard deviations of the ankle, shank, and knee segments are 1.2°, 4.5°, and 4.5° (Fig. 8). On rough terrain, the average standard deviations of ankle, shank, and knee were 4.1°, 9.6°, and 7.3°, respectively (Fig. 8c, e). This represents a 241% increase in variation in the ankle compared to 53% in the shank and 38% in the knee. To further compare the variance in angle between the three segments, we can represent the average standard deviation on rough terrain as a percentage of the total range of motion during level ground walking. By this metric, the ankle varies by 42% of its normal range of motion while the shank and knee vary by 11% and 12% of their normal ranges of motion, respectively. Furthermore, it is visually apparent that ankle adaptation occurs throughout the entirety of stance because the joint plantarflexes and dorsiflexes to match the terrain as soon as the foot contacts the ground. In contrast, the shank and knee trajectories on rough terrain are very similar to their respective level ground walking trajectories until about 50% of stride. Most modulation in the shank and knee positions occurs shortly before and after TO. The three amputee users were able to walk smoothly on rough, uneven terrain as the prosthesis allowed a greater range of motion in the ankle to adapt to the variability of the terrain.

#### C. Stair Ascent

**Fig. 9.**
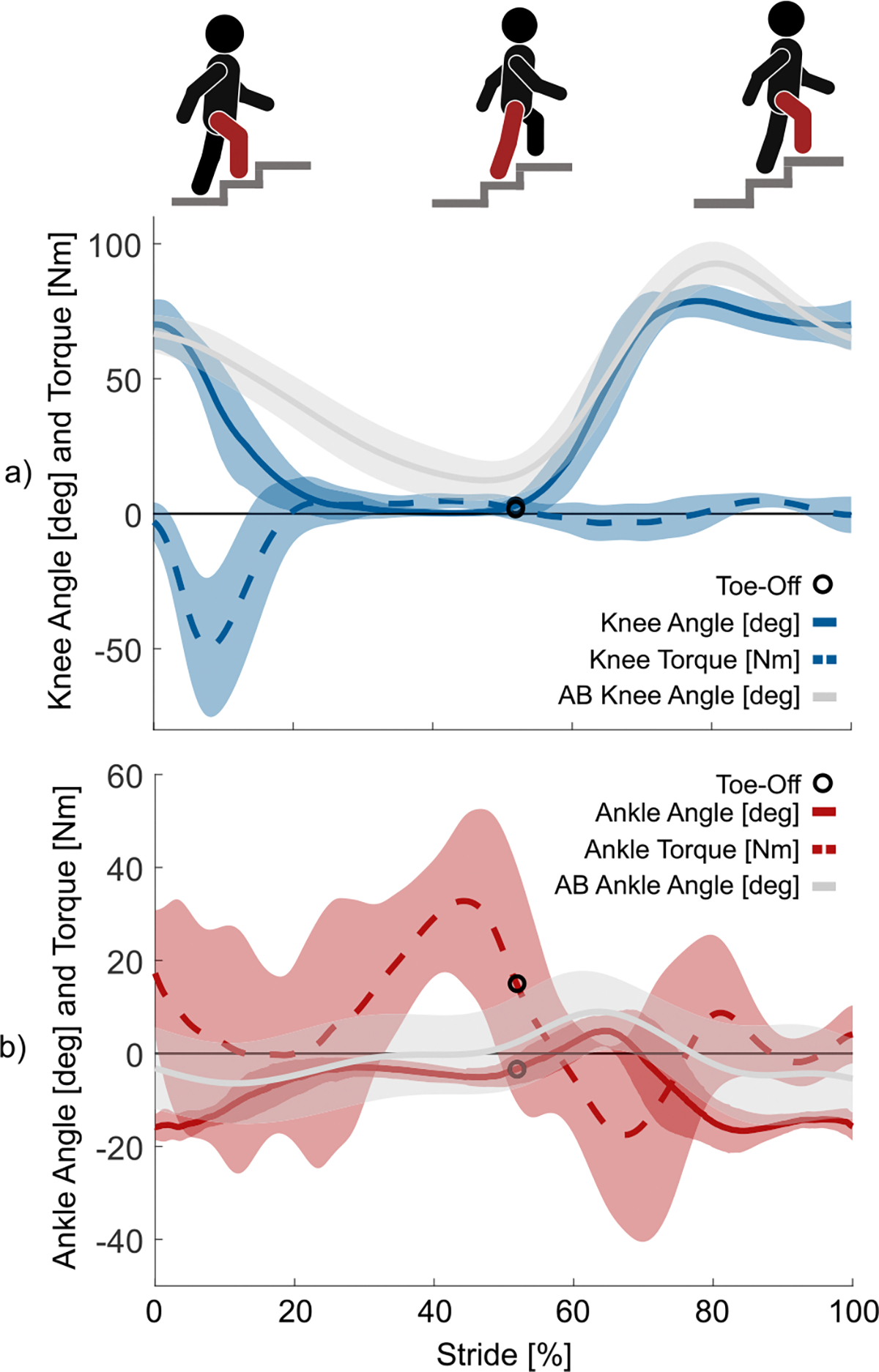
Stair ascent experiment. (a) Knee angle and torque and (b) ankle angle and torque of the prosthesis and AB reference during a single stair ascent stride. Data represents 30 total strides from three subjects Solid and dashed lines represent mean values ± one standard deviation as the shaded region. Able-bodied (AB) reference data is from 50 total strides across five subjects from Carmago et al. [66].

All three amputee users were able to perform biomimetic stair ascent. Fig. 9 shows joint angle and torque data from three amputee subjects ascending a uniform set of stairs. HS and TO events are determined by the GRF sensor described in IIA. HS and TO events from AB data are determined by motion capture. At 0% of stride, the controller commanded high knee flexion of 70.1+/− 9.2° (Fig. 9a). This starting position matches the knee angle at 0% stride in able-bodied populations of 66.7 +/− 6.9° (Fig. 9a). At the same time, the unified controller commanded ankle dorsiflexion of −15.8 +/− 2.9° to enable stable, flat foot placement on the stair (Fig. 9b). The ankle then plantarflexed as the knee extended and the prosthesis lifted the user up the stair between 0–30% of the stride. The knee commanded 49.3+/− 25.8 Nm of extension torque to enable the movement (Fig. 9a). This process occurred noticeably faster than the able-bodied reference, however users reported that the speed of knee extension was comfortable (Fig. 9a). At toe-off, the knee and ankle joints were close to neutral positions, similar to able-bodied kinematics (Fig. 9). During early swing, between 50–70% of the stride, the controller commanded knee flexion proportional to the user’s residual thigh kinematics. This control enabled rapid flexion to clear the previous stair (Fig. 9a). Similar to able-bodied kinematics, the ankle plantarflexed during this period to avoid scuffing on the stair (Fig. 9b). In late swing, the knee remained flexed and the controller commanded ankle dorsiflexion to prepare for stable foot placement on the next stair. The three amputee users were able to ascend stairs as the prosthesis commanded high extension torque and biomimetic joint kinematics.

### Transition Activities

#### D. Sit to Stand

**Fig. 10.**
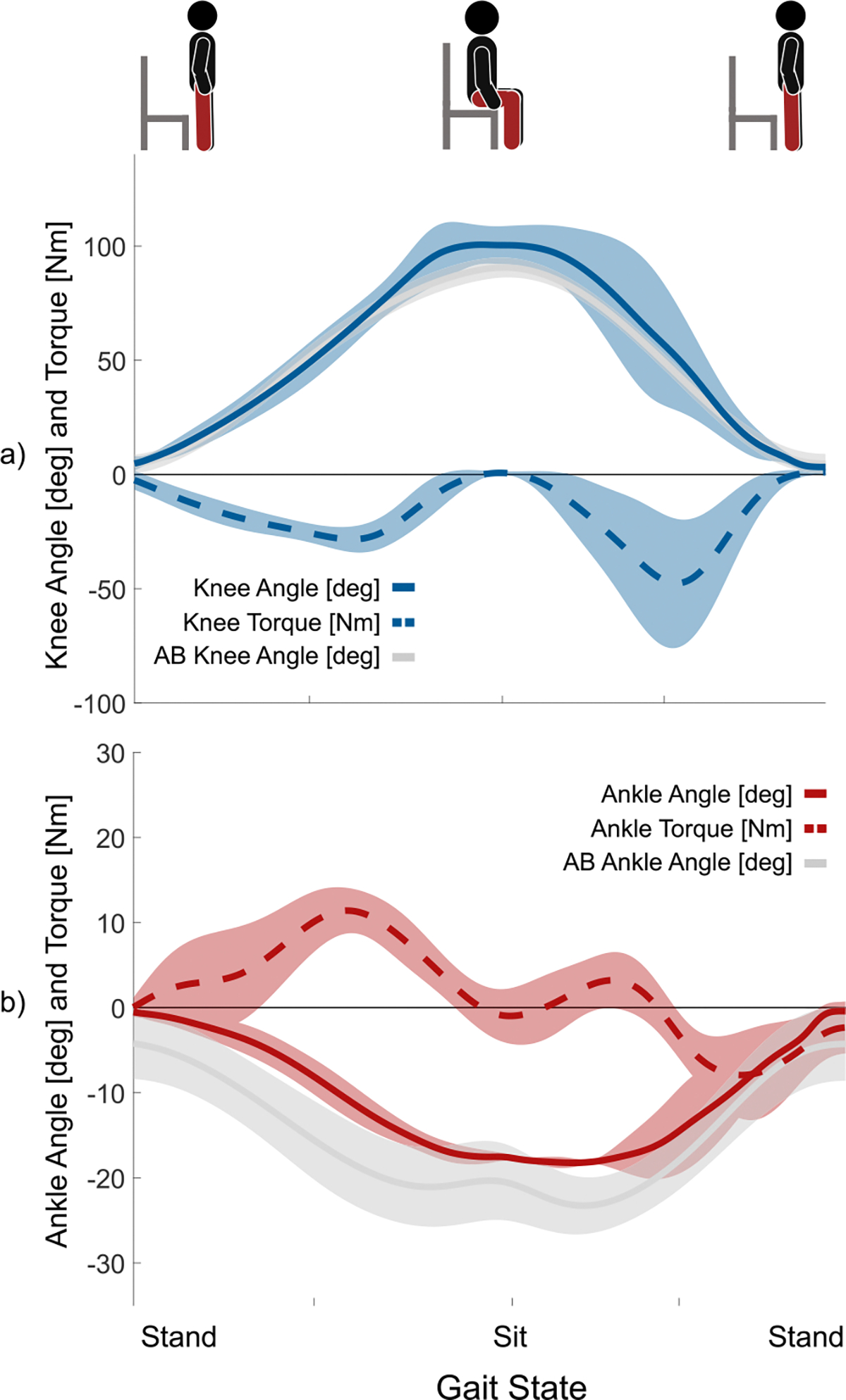
Sit to Stand experiment. (a) Knee angle and torque and (b) ankle angle and torque of the prosthesis and AB reference during a stand-sit-stand transition sequence. Data represents 9 total trials across three subjects Solid and dashed lines represent mean values ± one standard deviation as the shaded region. Able-bodied (AB) reference data is from 50 total strides across five subjects from Reznick et al. [67].

All three amputee users were able to perform natural stand-to-sit and sit-to-stand movements. Fig. 10 shows joint angle and torque data from three amputee subjects performing a continuous stand-sit-stand transition. Sit and stand events from AB data are determined by motion capture. During stand-to-sit, knee damping enabled the user to slowly perform the sitting movement at a controllable rate. These kinematics reasonably matched able-bodied reference data. In the sitting position, the peak knee angle from the average of our three test subjects was 100.9 +/− 7.9° compared to the able-bodied average of 91.9 +/− 4.8° (Fig. 10a). The ankle biomimetically dorsiflexed as the user sat down, and at the point of peak knee angle the ankle angle was an average of −17.6 +/− 0.7° compared to the able-bodied average of −19.4 +/− 4.7° (Fig. 10b). During sit-to-stand, the knee commands torque to enable the subjects to stand up quickly and controllably. Knee angle reasonably matched able-bodied reference data during this phase (Fig. 10a). The prosthesis biomimetically plantarflexed the ankle back to near 0° as the knee reached full extension (Fig. 10b). The three amputee users successfully performed stand-to-sit and sit-to-stand movements as the prosthesis provided positive and negative assistance and enforced biomimetic joint relationships.

#### E. Walk to Stairs Transition

**Fig. 11.**
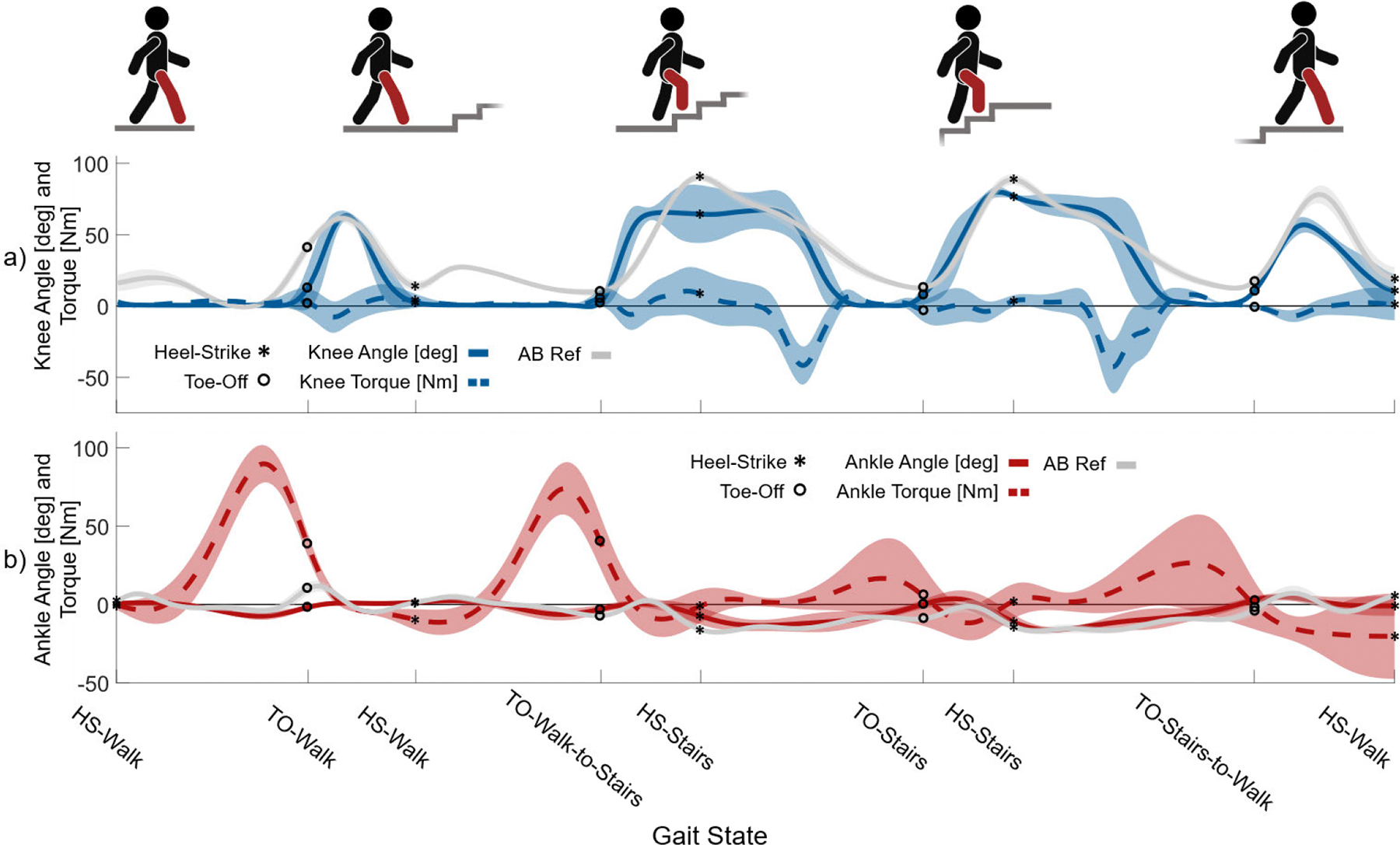
Walk-Stair Ascent-Walk experiment showing data from 9 total strides across three subjects. (a) Knee angle and torque, (b) ankle angle and torque during a walk-stair ascent-walk transition sequence containing four strides: level ground walking, walking to stairs transition, stair ascent, stairs to walking transition. In all subfigures, solid lines represent mean values and the shaded region represents ± one standard deviation. Able-bodied (AB) reference data is from 50 total strides, 10 strides each from five subjects, from Carmago et al. [66].

All three amputee users were able to perform natural transitions between walking and stair ascent, leading with the prosthesis. Fig. 11 shows knee and ankle joint position and torque data from three amputee subjects performing a continuous walk-stair ascent-walk transition. HS and TO events of the prosthesis are determined by the GRF sensor described in IIA. HS and TO events from AB data are determined by motion capture, and AB data were interpolated to align with the prosthesis timing. The sequence consisted of one full level ground walking stride, one transition stride from walk-to-stairs, one full stair ascent stride, and one transition stride from stairs-to-walk. The average prosthesis knee angle is shown in Fig. 11a. The knee angle trajectory, peak values, and HS and TO points aligned with able-bodied reference kinematics following an identical sequence. The prosthesis first enabled a level ground walking stride identical to those performed in previous experiments (Fig. 7). The peak prosthesis knee angle during walking was 64.0 +/− 3.1° compared to an able-bodied reference of 61.7 +/− 3.0°. Notably, the amputee users did not initiate knee flexion early in stance compared to able-bodied flexion of up to 19.8 +/− 5.5°. During the second stride, the users transitioned from walking to stairs. As the users prepared for foot placement on the first step, the prosthesis knee flexed up to 67.1 +/− 13.0° while the able-bodied average reached 90.3 +/− 2.6°. Despite the prosthesis knee angle being smaller than the AB reference in swing, all participants were able to clear the steps without additional compensation, as shown in the attached video. On the third stride, as the users performed a complete stair ascent stride, the knee angle peaked at 80.0 +/− 5.3° and the able-bodied reference peaked at 88.8 +/− 3.7°. Finally, when transitioning back to walking, the knee flexed during swing to 57.2 +/− 4.9° and the able-bodied reference reached 78.4 +/− 6.3°. To further compare the difference between the prosthesis and the able-bodied knee position during the sequence, we calculate the average difference between the two knee angles. The average difference in knee angle between the prosthesis and able-bodied data was just 12.8°. The three amputee users were able to transition between walking and stair ascent as the knee prosthesis biomimetically adapted behavior between the two activities, reasonably matching able-bodied kinematics.

The average prosthesis ankle angle is shown in Fig. 11b as the joint’s dorsiflexion and plantarflexion peaks align with the able-bodied reference kinematics. During the walking stride, the ankle dorsiflexed up to −9.0 +/− 3.1°, peaking at the same time as the able-bodied peak of −4.0 +/− 2.3°. The able-bodied ankle reference plantarflexed up to 11.7 +/− 3.3° after TO. In the transition from walking to stairs, the ankle reached peak dorsiflexion of −6.3 +/− 3.9° and peak plantarflexion after TO of 0.5 +/− 1.8. This closely matches the able-bodied reference, which dorsiflexed to −7.2 +/− 1.03° and plantarflexed to 2.5 +/− 2.2°. At HS-Stairs, the ankle dorsiflexed to −13.3 +/− 2.8° at HS and plantarflexed to 0.6 +/− 3.0° at TO. The able-bodied reference dorsiflexed to −17.7 +/− 1.3° and plantarflexed up to −9.1 +/− 1.4° at TO. Following the final TO event transitioning from stairs to walking, the ankle and able-bodied reference reached peak plantarflexion angles of 4.8 +/− 2.5° and 7.2 +/− 4.8°, respectively. The average difference in ankle angle between the prosthesis and able-bodied data across the entire sequence is 5.8°. The ankle prosthesis further enabled users to biomimetically transition between walking and stair ascent.

#### F. Real-World Ambulation

**Fig. 12.**
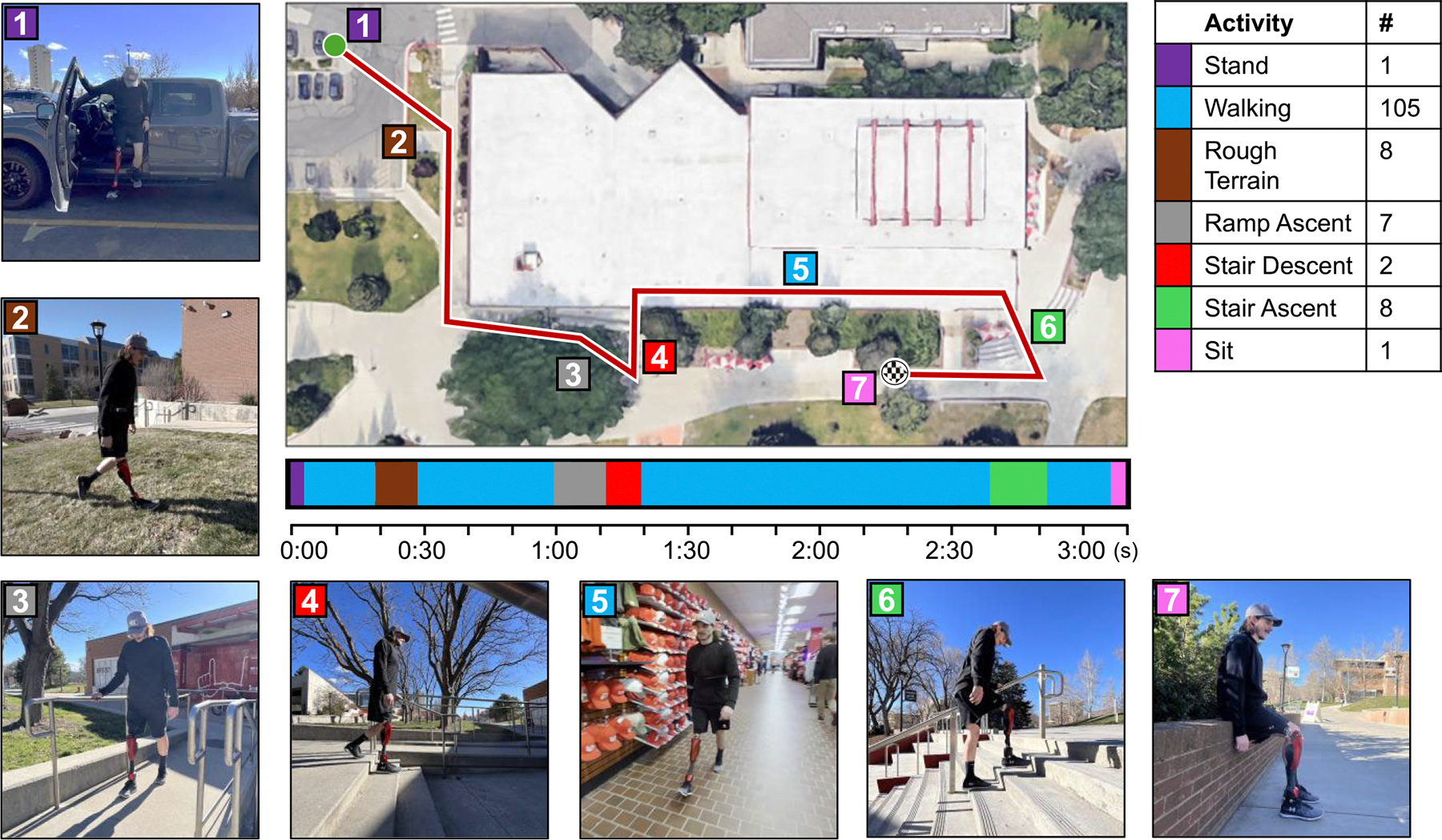
Real-World Ambulation Test. A top-down view of the University of Utah campus store shows a map of the test route. Numbered and color-coded tags correspond to the photos displayed alongside the map, showing the subject performing each of the different activities. A timeline below the map displays the type, timing, and duration of activities performed during the 3 minute and 11 second test. The table displays the types of activities, their corresponding color code, and the number of strides of each activity that were performed during the test.

One subject conducted a real-world ambulation test, performing a variety of activities and transitions in a continuous sequence. Fig. 12 shows a map of the experiment circuit performed by TF01 with pictures from different activities, a timeline of the test with color-coded activities, and a table of the activities performed as well as how many strides of each activity were performed. A video of the experiment is provided in the supplementary material of this paper. Prior to beginning the experiment, the subject practiced walking, ramp ascent, sit-to-stand, stair ascent, and stair descent in a laboratory setting. Experimenters tuned the eight parameters for subject preference, specifically adjusting the range of walking swing duration, maximum torque during stair ascent, knee flexion damping coefficient, and ankle stiffness coefficients. The subject was instructed to follow a pre-determined course through the University of Utah campus store, where a variety of ambulation activities could be performed in a short sequence, and they practiced the course three times before we recorded the experiment. During the course, the subject exits a car, traverses a parking lot, walks on uneven ground, ramps, and through a store, and performs stair ascent and descent. The prosthesis enabled the subject to perform all these activities and transitions between them by continuously adapting behavior based on the subject’s kinematics and interaction with the environment.

The subject successfully performed a high volume and variety of ambulation activities and transitions safely and independently. During the test, the subject took 105 level ground walking strides. They walked at variable speeds, moving faster across open spaces such as the parking lot and slower when constrained by obstacles and other shoppers within the store. Of the walking strides, 12 were irregular “shuffle” steps as they subject browsed items in the store or stepped backwards to open doors. The subject performed eight walking strides on rough terrain as they crossed an uneven grassy slope and seven strides ascending ramps. The ankle prosthesis enabled the subject to walk smoothly across the grass without adjusting their course or gait to compensate. Ankle adaptation furthermore enabled the subject to walk smoothly and balanced up the ramp. The subject performed eight stair ascent strides, receiving adequate foot clearance over each step and sufficient power to climb stairs with one handrail. The subject performed two stair descent strides, receiving high damping to descend slowly and safely. The subject performed one sit-to-stand and one stand-to-sit activity at the beginning and end of the test, respectively. The prosthesis enabled these steady-state activities as well as nine transitions between the activities. The proposed controller continuously adapted the prosthesis’s behavior in response to changes in the subject’s kinematics as they encountered unique terrain and required unique assistance. The duration of the test was three minutes and 11 seconds, during which the subject completed the experiment without any instructions or feedback from the experimenters. No parameters, modes, or other controls were altered during the test. The subject did not scuff their foot, trip, or experience unexpected behavior from the prosthesis at any point during the test. The prosthesis enabled the subject to ambulate in a real-world environment, utilizing the proposed unified control strategy to adapt to the needs of the subject and environment without classifying the desired activity.

## IV. Discussion

### A. Significance

This paper presents a unified controller for powered knee and ankle prostheses. The unified controller incorporates bioinspired behaviors to achieve natural adaptation and continuous transitions between activities. In this work, we show that this is possible without assuming periodicity or explicitly classifying activities. Instead, the controller modulates behavior as a continuous function of the prosthesis and user kinematics. This approach enabled three above-knee amputee participants in this study to walk on level, inclined, and uneven terrains, ascend and descend stairs, stand up and sit down from a chair, and seamlessly transition between all these activities while leading with their sound or prosthesis side. To the best of our knowledge, this is the first successful implementation of a unified control strategy for continuous ambulation across these activities for powered knee and ankle prostheses.

The proposed control approach diverges from the widespread assumption of periodicity in powered prosthesis control. Virtually all available powered prosthesis controllers define the prosthesis movements based on a periodic gait cycle specific to a certain ambulation activity or transition. As a consequence, they must first correctly classify the activity intended by the user to function properly. Even with up to 98% classification accuracy by the most sophisticated existing methods, an amputee user performing hundreds of transitions and thousands of steps per day will frequently encounter classification errors [48]. Real-world ambulation requires prosthesis users to quickly start, stop, change speed, change terrain, or change activity instantly. These movements are not periodic and are difficult to classify as they are highly variable and do not match activity-specific steady-state kinematics/kinetics profiles. The proposed controller enables aperiodic movements that are not classified as a specific activity by relying on biological gait behaviors that are invariant across ambulation tasks (e.g., minimum-jerk movements, knee/ankle synergy) and can operate simultaneously without interfering with each other.

The proposed controller builds logically on our previous work [68], [69]. To achieve adaptation to speeds and inclines, we use variable impedance control for the ankle joint. Ankle stiffness is commanded as a function of shank angle, a control approach originally developed and validated for transtibial users with a semi-active ankle prosthesis [54]. In this study, we demonstrate the first successful implementation of shank-based stiffness for above-knee amputees. More importantly, in this study, we extended this shank-based approach to late stance, showing that it enables the ankle to adapt energy injection to variable inclines. Moreover, in this study, we present a novel control approach that commands ankle damping as a continuous function of the user’s hip velocity. This novel variable-damping approach enables the prosthesis to provide higher positive power output at the ankle as walking speeds increase. Thus, this work expands shank-based impedance control to allow for variable energy injection for different speeds and inclines.

Speed and incline adaptation for powered knee and ankle prostheses has been achieved in previous studies using different control approaches. One approach for speed adaptation uses independent sub-controllers for each selected speed and requires hand-tuned impedance parameters for each speed [70]. This approach is highly functional within discrete cases; however, it does not enable continuous adaptation. A second approach for speed adaptation enforces quasi-stiffness profiles extracted from nominal biomechanics, determining the appropriate profile using the prosthesis kinematics and an estimate of ambulation speed [27]. This approach enables continuous adaptation but relies on lookup tables that have saturation limits outside the table boundaries, which limits versatility and requires a walking speed estimate. A third approach achieves data-driven variable impedance for adaptive speed and incline walking by optimizing impedance parameters based on an estimate of gait phase, speed, and incline [26]. This approach improves continuous adaptation but remains reliant on accurate estimates of gait characteristics and enforces nominal biomechanics, which may be challenging to adapt to the subjective preference of different amputee users and their clinicians. Different from these previous approaches, the proposed controller continuously adapts its speed as a function of the user’s hip velocity and adapts its incline solely as a function of the shank angle. This approach provides adaptation based on measurable variables that are not defined by gait phase and do not require periodic ambulation. Furthermore, it allows for adaptation to rough terrains, a feature not demonstrated with other powered knee and ankle controllers.

As shank-based stiffness commands ankle torque in late stance, we implement a novel control approach that reproduces the function of the biarticular gastrocnemius muscle. This biarticular term commands flexion torque at the knee proportional to ankle plantarflexion torque, which initiates swing in late stance, and injects energy at the knee joint in late stance. Thus, by continuously modulating the knee flexion torque as a function of the ankle torque and knee position, this biarticular term allows for swing initiation and energy injection at the knee joint without estimating the gait phase or classification of the activity.

To achieve speed adaptation during swing, we present a novel implementation of minimum-jerk trajectory control. This novel implementation enables instantaneous speed adaptation without using gait phase or assuming periodicity. In previous studies, the duration of the minimum jerk trajectory was calculated proportional to the duration of the previous stance phase, essentially relying on the assumption that the movement is periodic. Moreover, the swing was split into two phases (flexion/extension), and the maximum flexion position was either pre-determined [56] or commanded based on thigh position [53]. These characteristics made the swing controller activity specific. In this study, we expanded the minimum-jerk control, commanding a single movement with no predetermined maximum flexion angle. Furthermore, we calculate the swing duration based on the ankle angle at toe off. This relationship between swing duration and ankle angle at toe off is inspired by able-bodied biomechanics and enables the user to change speed instantly.

During the walking experiments, the prosthesis biomimetically adapted behavior to enable natural ambulation on variable inclines, at variable speeds, and on rough terrain. When walking at variable inclines, the ankle prosthesis closely matched able-bodied trends by increasing dorsiflexion and positive energy injection as the ramp incline increased. Meanwhile, the knee prosthesis trajectory remained consistent and stable across inclines. When walking at variable speeds, the ankle prosthesis closely matched able-bodied trends by decreasing damping and therefore increasing positive energy injected as walking speed increased. Triggered by the ankle’s adaptation to walking speed, the duration of the knee swing trajectory decreased as walking speed increased, so that both swing and stance phases of walking decreased biomimetically. Importantly, the prosthesis is not discretely tuned for specific inclines or speeds. Instead, the controller automatically and continuously adapts behavior in response to the user changing their gait behavior. When walking on rough, uneven terrain, the ankle range of motion increased substantially to adapt to the highly inclined and declined surfaces. The knee and shank, in contrast, moved more consistently between rough and level terrain. These experiments show that the controller naturally adapts to changes in speed, incline, and terrain type. This approach allows users to safely encounter variable terrain types and walk at variable speeds necessary for ambulation outside the laboratory setting.

The proposed Step-up Torque was originally developed for a previous stair-ascent controller and validated with one amputee subject [52]. This paper further validates this approach with three amputee participants. When performing stair ascent, the Step-up Torque enabled all three participants to climb stairs smoothly and stably. This work presents a novel implementation of this control function by enabling Step-up Torque to support both stair ascent and sit-to-stand transitions without interfering with other ambulation activities, which is key to avoiding the need to classify the intended activity. When performing sit-to-stand transitions with the Unified Controller, the users’ kinematics mirrored stair ascent conditions. As a result, the controller commanded Step-up Torque to support smooth and powerful sit-to-stand.

Similar to our previous work, we implement synergy-based desired position control [52] and minimum-jerk control [53], [56], [71]. However, here we present a novel implementation that integrates these two control strategies, extending their capability to multiple ambulation activities. Specifically, the proposed controller determines the initial prosthesis swing behavior based on the kinematics of the residual thigh at toe off and continuously adapts them throughout swing. This continuous adaptation enables the powered prosthesis to support both walking and stair climbing under a single controller. Furthermore, users can transition between walking and stairs leading with either the prosthesis or the intact limb. During the complex walk-stairs-walk transitions performed here, the prosthesis successfully modulated its behavior over the course of a single stride to enable the user to seamlessly transition between the two activities. Notably, transitions between walking and stair ascent have been achieved by varying prosthesis behavior between two data-driven, steady-state controllers in results from [25] as well as [72]. Those continuously varying approaches were validated to be more beneficial than simple mode-switching approaches and were shown to greatly improve biomimicry of activity transitions. However, the approaches still require the controller to correctly classify the activity and intended transition.

The ultimate experiment in this work was to perform all the previously tested activities and transitions, as well as additional, untested environments and combinations of transitions, in a real-world environment. The prosthesis enabled smooth and safe ambulation during all activities performed during the test, including walking on new terrain types and changing speeds based on moving obstacles. The user encountered unpredictable elements of real-world environments, and the prosthesis adapted accordingly. Furthermore, the prosthesis enabled seamless transitions without classifying the desired starting and ending activities, performing each transition as soon as the user required it. This capability substantially improves the real-world viability of powered prostheses.

### B. Limitations

We recognize several limitations in the approach and capabilities of the proposed control strategy. First, the proposed controller does not use EMG sensors to provide the user with volitional control over the prosthesis’ movement. Volitional control ensures that prosthesis assistance is coordinated with user intent. This ability makes EMG-based control a potential alternative approach to achieving ambulation without activity classification [16], [73]. Several existing controllers have demonstrated EMG control for sit-to-stand, walking, squatting, lunging, and postural control [41], [51], [74], [75]. Future work could address the challenges of implementing EMG sensors into the prosthetic socket and develop a unified controller using EMG input. We believe that this approach could be preferred by users and improve adaptability in real-world scenarios.

The unified controller does not reproduce able-bodied biomechanics in all scenarios. During level ground walking, damping in the knee joint prevents the user from initiating the common normative behavior of stance knee flexion. Users did not note this as a significant limitation. This is likely because amputees are often trained to walk on passive knee prostheses without stance knee flexion.

The experiments presented here demonstrate the functionality of the controller and compare basic biomechanics to able-bodied references. However, they do not assess whether the proposed controller achieves optimal, biomimetic ambulation, as done in previous studies [18], [26], [50], [62]. Although subject-specific tuning is not required for the controller to function, we did tune up to eight control parameters to the liking of the three participants. During the subject’s 30-minute practice period, tuning the parameters took about 15 minutes, which is similar to the time spent by a prosthetist to tune passive microprocessor-controlled prostheses. However, properly tuning the parameters requires specific expertise and experience with the proposed controller, limiting clinical viability. Despite this, we believe that the ability to tune the controller is an advantage because it enables us to account for subjective user and clinician preferences. In our previous work [27], we scaled all control parameters based on body weight using nonamputee biomechanics as a reference. This approach led to a fundamentally tuning-free walking controller [27]. However, it would not allow us to implement subjective preference. Future work should make this process more intuitive and accessible, allowing a subject to tune the prosthesis without direct supervision of clinicians or engineers [23]. This could be achieved by reducing the number of parameters. Alternatively, future work could use optimization algorithms or automatic tuning to select parameters, as previously done in [76], [77], and [78]. Additionally, the controller’s generalizability may be limited by the amount of training time required for different users, depending on their intuitive behaviors and the expertise of the trainer.

Specific limitations also exist in the implementation of the proposed controller. Ankle damping is commanded as a function of hip velocity and saturates at 0 Nm*s/deg at 1.2 m/s in plantarflexion and 2.0 m/s in dorsiflexion. Participants in this study did not reach hip velocities greater than 1.2 m/s. However, future users who may be able to reach higher hip velocities will not receive continuous adaptation above this speed. The minimum jerk trajectory duration adaptation is additionally limited to a range of 0.45–0.55 s. Participants in this study did not make note of this impacting their ability to walk at the fastest and slowest tested speeds. However, this saturation may limit versatility for future users. Knee damping in the flexion direction is commanded when the thigh is anterior to the body, which occurs during both stair descent and stand-to-sit. Therefore, the prosthesis commands equivalent damping in both activities. Participants in this study did not report any difficulty when performing the stand-to-sit. However, future users may prefer to receive higher damping during stair ascent when lowering on one leg and lower damping during stand-to-sit when lowering on both legs. An alternative control approach to address this limitation would be to command knee damping as a function of GRF magnitude. In stair descent, the controller does not command a minimum jerk trajectory but instead is position controlled to return to 2° with loose PID gains. Participants in this study were able to safely descend stairs and did not note difficulty with this control.

## V. Conclusion

In this work, we present a novel unified control strategy for a powered knee and ankle prosthesis. The unified controller is intrinsically adaptive to the user and environment, enables a variety of ambulation activities, and supports seamless transitions between activities without classifying the desired activity or assuming a periodic gait. The controller builds upon the successes of previous control strategies and incorporates biologically inspired gait behaviors to enable safe and natural ambulation.

Experiments with three above-knee amputees show that the proposed controller enables walking on level, inclined, and uneven terrains, ascending and descending stairs, and standing up and sitting down from a chair. The controller uses a bioinspired approach to replicate able-bodied behavior during the tested activities. This represents the first implementation of a powered knee and ankle prosthesis controller to achieve this level of functionality and versatility. Furthermore, users were able to smoothly transition between all these activities. The prosthesis kinematics closely matched able-bodied reference data during sit-to-stand, stand-to-sit, walk-to-stair ascent, and stair ascent-to-walk. Finally, one subject performed a real-world ambulation experiment in an uncontrolled, mixed outdoor and indoor environment. The prosthesis enabled safe and continuous ambulation across a variety of activities as the user encountered obstacles, navigated previously untested terrain, and moved between tasks as they desired.

The unified controller enables lower-limb amputees to use powered prostheses and ambulate safely and naturally. We believe that this work represents a promising advancement in the translation of powered prostheses from the laboratory to the real world.

## Supplementary Material

supp1-3595496

supp2-3595496

This article has supplementary downloadable material available at https://doi.org/10.1109/TNSRE.2025.3595496, provided by the authors. Digital Object Identifier 10.1109/TNSRE.2025.3595496

## Figures and Tables

**TABLE I:** Subject Information

Subject Code	Sex	Age [years]	Mass [kg]	Height [m]	Amputation Side	Time Since Amputation [years]	Suspension Type
TF01	M	30	65	1.78	R	10	Suction
TF02	F	34	59	1.60	L	14	Lanyard
TF03	M	55	78	1.72	L	3	Suction
