# Supplementary material for: Unified Control of a Powered Knee-Ankle Prosthesis Enables Walking, Stairs, Transitions, and Other Daily Ambulation Activities
Source: IEEE Trans Neural Syst Rehabil Eng. Author manuscript; Available in PMC 2025 Sep 19. (PMC12447845; doi:10.1109/TNSRE.2025.3595496)
Supplement: supp1-3595496 [file NIHMS2104034-supplement-supp1-3595496.docx]

Supplementary Materials for:

Unified control of a powered knee-ankle prosthesis enables walking, stairs, transitions, and other daily ambulation activities.

Sullivan et al.

Contents:

I. SUPPLEMENTARY METHODS

S1, Virtual Damping Control

*S1a, Knee Damping*

*S1b, Ankle Damping*

S2, Joint Synergy Control

*S2a, Knee Synergy*

*S2b, Ankle Synergy*

II. SUPPLEMENTARY RESULTS

S3, Walking

S4, Variable Speed Walking

S5, Stair Ascent

S6, Rough Terrain

S7, Sit to Stand

S8, Walk-Stair Ascent-Walk Transitions

*S8i, Walk-Stair Ascent-Walk PSF*

*S8ii, Walk-Stair Ascent-Walk SSF*

*S8iii, Walk-Stair Descent-Walk PSF*

*S8iv, Walk-Stair Descent-Walk SSF*

# I. SUPPLEMENTARY METHODS

S1, Virtual Damping Control

*S1a, Knee Damping*

Virtual knee damping is used to stabilize and smooth the movement of the prosthesis during all activities. Following (S1), $T_{Knee}^{Damping}$ is defined as the negated product of a virtual damping coefficient ($B^{Knee}$) and the measured knee velocity ($\dot{\theta}_{Knee}^{\mathrm{Meas}}$). $B^{Knee}$ was tuned in this work for user preference. Distinct damping coefficient values are used for extension ($\dot{\theta}_{Knee}^{\mathrm{Meas}}<0$) and flexion ($\dot{\theta}_{Knee}^{\mathrm{Meas}}\geq0$), following (S2).

$$\begin{aligned} T_{Knee}^{Damping}=-\left( B^{Knee}\cdot\dot{\theta}_{Knee}^{Meas} \right)\#\left( S1 \right) \end{aligned}$$

$$\begin{aligned} B^{Knee}=\left\{ \begin{aligned} B^{Ext}, if \dot{\theta}_{Knee}^{\mathrm{Meas}}<0 \\ &B^{Flex}, if \dot{\theta}_{Knee}^{\mathrm{Meas}}\geq0 \end{aligned} \right.\#\left( S2 \right) \end{aligned}$$

The damping coefficient during extension, $B^{Ext}$, is a function of the position of the knee. $B^{Ext}$ has a maximum value of 0.3 $\frac{Nm*s}{deg}$ when $\theta_{Knee}^{Meas}<$ 10° and decreases linearly to $0 \frac{Nm*s}{deg}$between 10° $<\theta_{Knee}^{Meas}<$20° (1d). $B^{Ext}$ allows the knee prosthesis to slow down before reaching the full-extension end-stop during extension movements like stair climbing and sit-to-stand transitions.

The damping coefficient during flexion, $B^{Flex}$, is a function of the global orientation of the residual thigh ($\theta_{Thigh}^{Meas}$), which is negative when the thigh is anterior to the torso and positive when posterior. $B^{Flex}$is at a maximum value of $0.5 \frac{Nm*s}{deg}$ when $\theta_{Thigh}^{Meas}<5^{\circ}$ and decreases linearly to $0 \frac{Nm*s}{deg}$between 5° $<\theta_{Thigh}^{Meas}<$10° (1e). $B^{Flex}$prevents the knee from buckling under the user’s weight when the prosthesis is in front of the user, such as in the early stance of walking. As the thigh moves posteriorly, $B^{Flex}$decreases to zero allowing the prosthetic knee to flex during swing initiation.

*S1b, Ankle Damping*

$T_{Ankle}^{Damping}$ is defined as proportional and opposite to ankle velocity ($\dot{\theta}_{Ankle}^{Meas}$) based on the ankle damping coefficient ($B^{Ankle}$) (S3). Distinct damping coefficients are used for dorsiflexion ($B^{DF}, \dot{\theta}_{Ankle}^{Meas}<0$) and plantarflexion ($B^{PF}, \dot{\theta}_{Ankle}^{Meas}\geq0$) (S4). $B^{Ankle}$was tuned in this work for user preference. Notably, when the prosthesis is in front of the body, plantarflexion damping is fixed to provide consistent behavior at HS. When $\theta_{Shank}^{Meas}$ < 0°, $B^{Ankle}$ is set to 1.3 (S4).

$$\begin{aligned} T_{Ankle}^{Damping}=-\left( B^{Ankle}*\dot{\theta}_{Ankle}^{Meas} \right)\#\left( S3 \right) \end{aligned}$$

$$\begin{aligned} B^{Ankle}=\left\{ \begin{aligned} 1.3, \mathrm{if} \theta_{Shank}^{Meas}<0 \\ B^{PF}, if \dot{\theta}_{Ankle}^{Meas}\geq0 \\ &B^{DF}, if \dot{\theta}_{Ankle}^{Meas}<0 \end{aligned} \right.\#\left( S4 \right) \end{aligned}$$

Both dorsiflexion damping $B^{DF}$ and $B^{PF}$ are functions of horizontal hip velocity $(v_{Hip}^{Meas}$), which is calculated online using the prosthesis joint positions and velocities following (S5) (Fig. S1).

$$\begin{aligned} \begin{matrix} v_{Hip}^{Meas}=(sin \left( \alpha+\theta_{Foot}^{Meas} \right)*l_{Foot}^{Meas}*\dot{\theta}_{Foot}^{Meas})+ \\ (cos \left( \theta_{Shank}^{Meas} \right)*l_{Shank}^{Meas}*\dot{\theta}_{Shank}^{Meas})+ \\ (cos \left( \theta_{Thigh}^{Meas} \right)*l_{Thigh}^{Meas}*\dot{\theta}_{Thigh}^{Meas}) \end{matrix}\#\left( S5 \right) \end{aligned}$$

As $v_{Hip}^{Meas}$ increases, $B^{PF}$ and $B^{DF}$ decrease (Fig. 3d). Specifically, $B^{PF}$decreases linearly from a maximum value of 1.3 at 0 m/s to 0 at 1.2 m/s (Fig. 3d), whereas $B^{DF}$ decreases from a maximum value of 1.2 at 0 m/s to 0 at 2.0 m/s (Fig 3e).


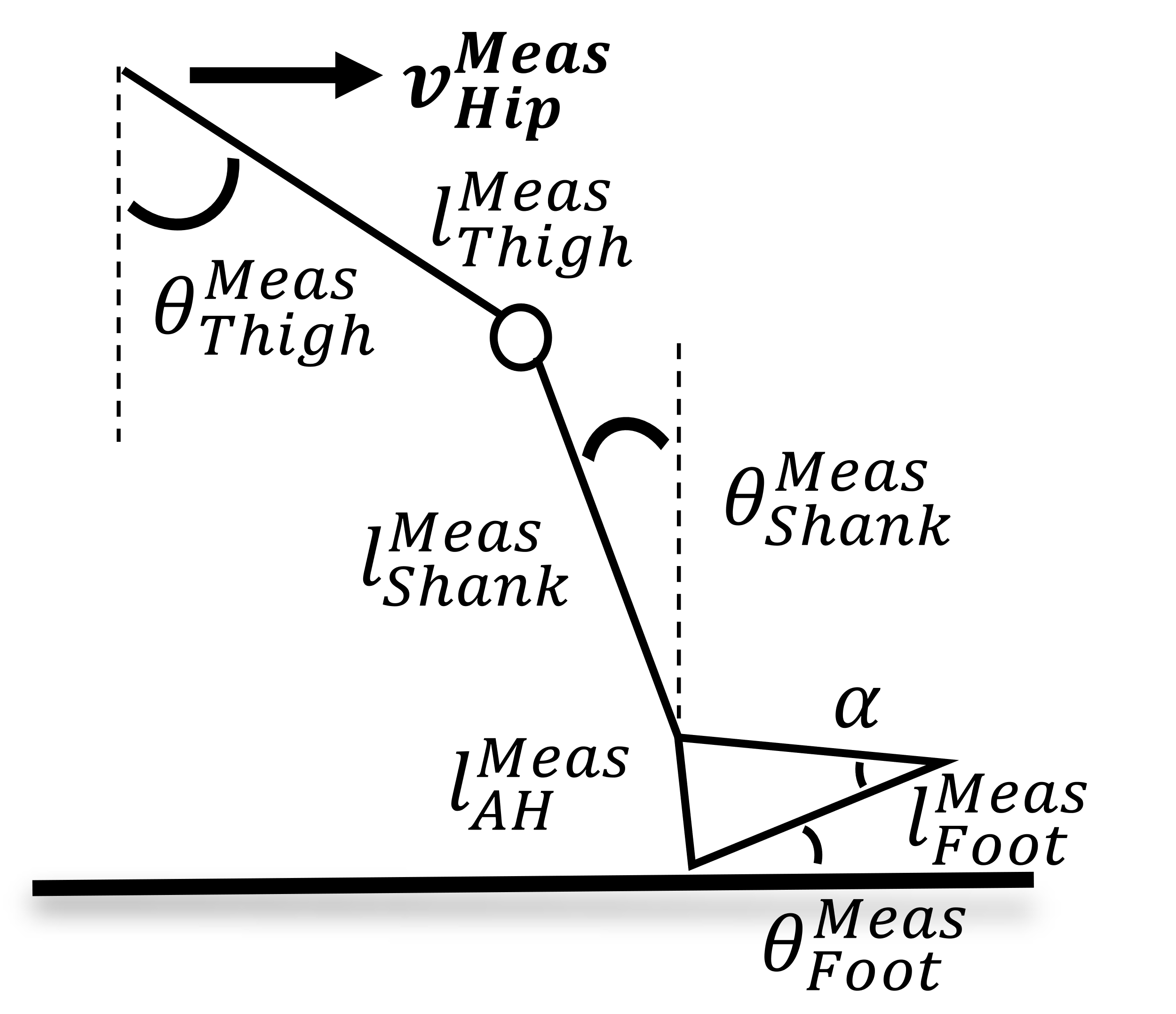


Fig. S1 Kinematic model of leg joint angles and segment lengths used to calculate horizontal hip velocity, $\boldsymbol{v}_{\boldsymbol{Hip}}^{\boldsymbol{Meas}}$.

S2, Joint Synergy Control

*S2a, Knee Synergy*

$\theta_{Knee}^{Syn}$ is defined as the sum of three terms (S6). The first term is the position-based synergy angle ($\theta_{Knee}^{Pos}$), which increases from 0° to 65° as the thigh flexes from 0$^{\circ}$ to -50° (Fig. S2a) (S7). Hard-coded angle parameters can be tuned for user preference. $\theta_{Knee}^{Syn}$ causes the knee to flex when the thigh flexes forward. The second term is the velocity-based synergy angle ($\theta_{Knee}^{Vel})$. This angle increases proportionally to thigh velocity ($\dot{\theta}_{Thigh}^{Meas}$) following a constant gain of 0.005 (S8) (Fig. S2b). As a result, the faster the thigh rotation, the greater the knee flexion angle. The third component is the acceleration-based synergy angle ($\theta_{Knee}^{Acc}$), which increases as a function of the vertical acceleration of the thigh segment ($\ddot{y}_{Thigh}^{Meas}$) (S9) (Fig. S2c). To compute $\theta_{Knee}^{Acc}$, we double integrate $\ddot{y}_{Thigh}^{Meas}$ and subtract a constant value of 1.03 to prevent integral windup, with respect to time. We then multiply the value by a constant gain of 2,500 and by a variable gain called synergy acceleration gain ($K^{Acc})$, which changes as function of $\theta_{Thigh}^{Meas}$ (Fig. S2d). The constant gain can be tuned for user preference. $K^{Acc}$ is equal to 1 when $\theta_{Thigh}^{Meas}>$ -20° and decreases linearly to 0 between -20° $>\theta_{Thigh}^{Meas}>$-50°. Following (S9), the knee flexion angle increases proportionally to the vertical displacement of the user's thigh. This movement is necessary to enable users to clear the trailing step when walking up the stairs step-over-step.

$$\begin{aligned} \theta_{Knee}^{Syn}= \theta_{Knee}^{Pos}+\theta_{Knee}^{Vel}+\theta_{Knee}^{Acc}\#\left( S6 \right) \end{aligned}$$

$$\begin{aligned} \theta_{Knee}^{Pos}=\left( \frac{65^{\circ}}{-50^{\circ}} \right)*\theta_{Thigh}^{Meas}\#\left( S7 \right) \end{aligned}$$

$$\begin{aligned} \theta_{\mathrm{Knee}}^{Vel}= 0.005*\dot{\theta}_{Thigh}^{Meas}\#\left( S8 \right) \end{aligned}$$

$$\begin{aligned} \theta_{\mathrm{Knee}}^{Acc}= K^{Acc}*2500*\iint(\ddot{y}_{Thigh}^{Meas}-1.03) dt\#\left( S9 \right) \end{aligned}$$

*S2b, Ankle Synergy*

$\theta_{Ankle}^{Syn}$ is defined as the sum of two terms. The first term is a position-based synergy ($\theta_{Ankle}^{Pos}$) and the second is an acceleration-based synergy ($\theta_{Ankle}^{Acc}$) similar to $\theta_{Knee}^{Acc}$ (S10). $\theta_{Ankle}^{Pos}$, which is proportional to $\theta^{AK}$, becomes more negative as the knee flexes (14, S11) (Fig. S2b). Thus, this term creates a knee flexion/ankle dorsiflexion synergy. $\theta_{Ankle}^{Acc}$ decreases proportionally to the vertical acceleration of the thigh (S12) (Fig. S2c), similar to the knee (23). Thus, the ankle plantarflexes when the user’s thigh moves vertically, which is necessary to improve clearance for stair ascent when using a step-over-step or a step-by-step gait pattern. Based on (16), when $K^{Syn}$ is 0, $\theta_{Ankle}^{Des}$ is 0° and the ankle is controlled by the loose PID controller to return to a neutral position.

$$\begin{aligned} \theta_{Ankle}^{Syn}=\theta_{Ankle}^{Pos}+\theta_{Ankle}^{Acc} \#\left( S10 \right) \end{aligned}$$

$$\begin{aligned} \theta_{Ankle}^{Pos}=K^{AK}*\theta^{AK}\#\left( S11 \right) \end{aligned}$$

$$\begin{aligned} \theta_{Ankle}^{Acc}= K^{Acc}*1000*\iint(\ddot{y}_{Thigh}^{Meas}-1.03) dt\#(S12) \end{aligned}$$

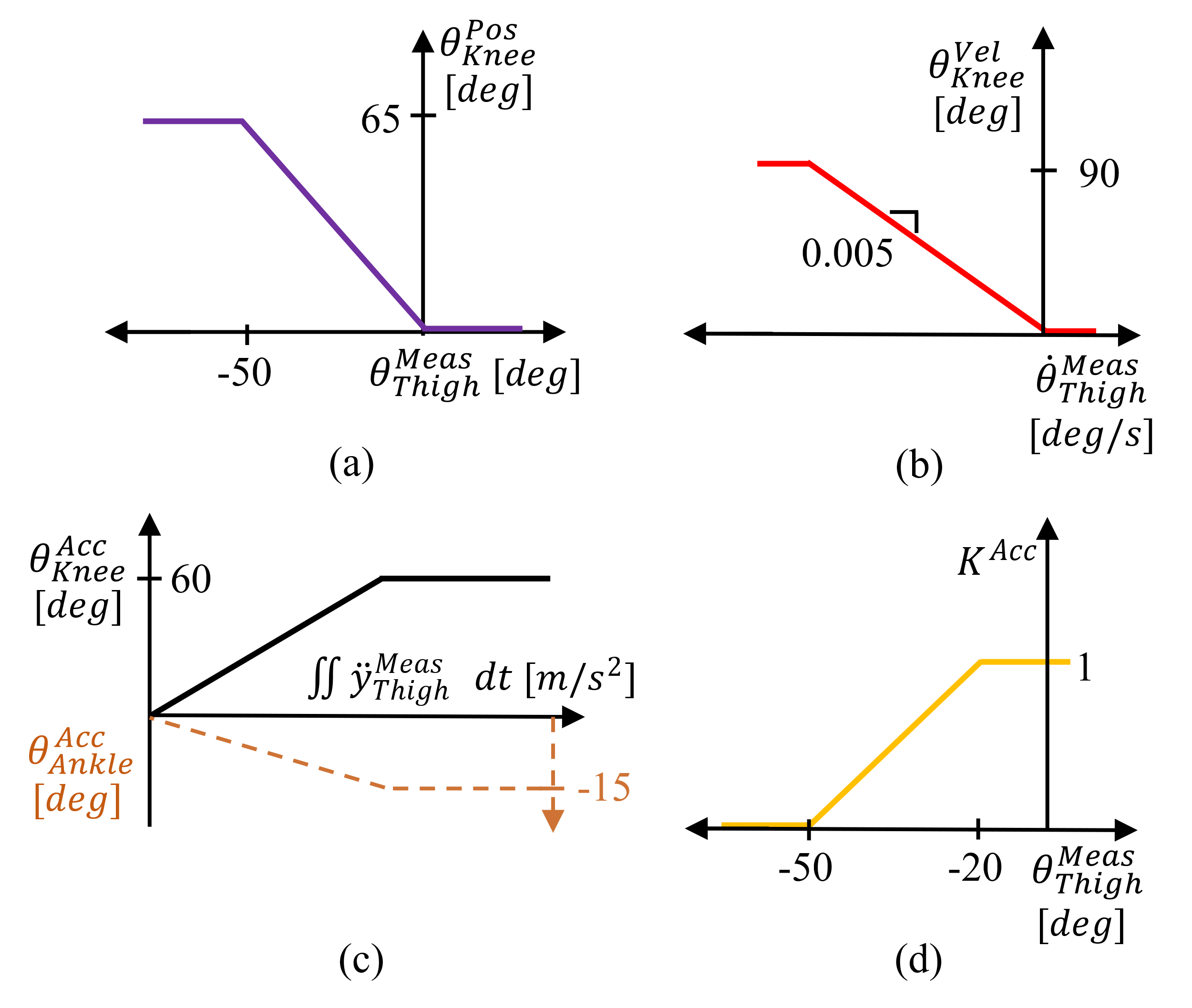


Fig. S2 Joint synergy control parameters. (a) Position-based, $\boldsymbol{\theta}_{\boldsymbol{Knee}}^{\boldsymbol{Pos}}$, and (b) velocity-based, $\boldsymbol{\theta}_{\boldsymbol{Knee}}^{\boldsymbol{Vel}}$, knee synergy angles. (c) Acceleration-based synergy angle for the knee, $\boldsymbol{\theta}_{\boldsymbol{Knee}}^{\boldsymbol{Acc}}$, and ankle, $\boldsymbol{\theta}_{\boldsymbol{Ankle}}^{\boldsymbol{Acc}}$, as a function of vertical thigh acceleration. (d) Acceleration gain, $\boldsymbol{K}^{\boldsymbol{Acc}}$, as a function of thigh angle.

II. SUPPLEMENTARY RESULTS

To demonstrate the functionality of the unified controller, the subjects first performed steady-state activities, including walking and stair ascent, to demonstrate the individual elements of the controller. Next, the subjects performed transition activities to show that the unified controller enables a variety of activities under a single controller. While all subjects completed all activities, each experiment figure presents data from a single subject to improve visual clarity, and the selected subject for each figure was chosen randomly.

Steady State Activities

S3, Walking

The unified controller enabled a stable stance phase during level ground walking. Fig. S3 shows joint position and torque data from TF02 during a stride from HS to HS while walking at a speed of 1.00 m/s on a zero-incline treadmill. The *Contact* control state begins at HS, or 0% of *Contact*, when the GRF sensor value increases above 120 N. In *Contact*, the unified controller calculates $T_{Knee}$ as the sum of $T_{Knee}^{Step-Up}$, $T_{Knee}^{Damping}$, and $T_{Knee}^{Biart}$ (1). $T_{Knee}^{Step-Up}$ commands 0 Nm throughout the *Contact* phase because the knee angle at HS$(\theta_{Knee}^{HS}$) is 1.52°, which is less than the preset end angle, $\theta_{Knee}^{End}$, of 5° (2-4) (Fig. 2a-b) (Fig. S3). $T_{Knee}^{Damping}$ commands up to 3.9 Nm of flexion torque between 0-20% of *Contact* as the user weights the prosthesis and the knee extends from 1.52° to 0° (7-8) (Fig. 2e) (Fig. S3). During this period of *Contact,* $T_{Knee}^{Damping}$ slows the movement of the knee and prevents high impact at the knee’s mechanical end stop. Additionally, low magnitude $T_{Knee}^{Damping}$ is commanded at around 50% and 75% of *Contact* as $\theta_{Knee}^{Meas}$ varies slightly (Fig. S3a). $T_{Knee}^{Damping}$ is zero as the knee flexes between 80-100% of *Contact* because the commanded damping coefficient, $B^{Flex}$, is set to 0 due to $\theta_{Thigh}^{Meas}$ being greater than 10° (S2) (Fig. 2e). $T_{Knee}^{Biart}$ commands up to 5 Nm beginning at 40% of *Contact*, remains constant until 80%, then decreases to 0 Nm between 80-100% (Fig. S3a). The modulation in $T_{Knee}^{Biart}$ is the result of its proportional relationship to $T_{Ankle}$ based on the variable gain $K^{Biart}$ (5). $T_{Ankle}$ is high as the user rolls over the foot between 40-80% of *Contact* (Fig. S3c). During the same period, $\theta_{Knee}^{Meas}$ is less than 20°, which sets $K^{Biart}$ equal to its maximum value and allows $T_{Knee}^{Biart}$ to increase (5) (Fig. 2c) (Fig. S3a). At 80% of *Contact*, the user unweights the prosthesis and $\theta_{Knee}^{Meas}$ begins to increase (Fig. S3a). As $\theta_{Knee}^{Meas}$ increases above 20°,$K^{Biart}$ decreases to zero, which causes $T_{Knee}^{Biart}$ to decrease to 0 Nm, avoiding excessive knee flexion (5) (Fig. 2c) (Fig. S3a). The *Contact* controller enabled a stable walking stance by utilizing knee kinematics and concurrent ankle kinetics to command desired knee torques.

The unified controller next enabled smooth swing during level ground walking. The *No Contact* control state begins at TO, or 0% of *No Contact*, when the GRF value decreases below 80 N. In *No Contact,* the unified controller defines a desired position, $\theta_{Knee}^{Des}$, as the sum of $\theta_{Knee}^{MJ}$ and $\theta_{Knee}^{Syn}$ weighted by a variable gain, $K^{Syn}$ (12). Throughout the *No Contact* phase of level ground walking, $\theta_{Knee}^{Syn}$ is equal to 0 and $\theta_{Knee}^{MJ}$ dominates $\theta_{Knee}^{Des}$ (Fig. S3b). $\theta_{Knee}^{MJ}$ dominates $\theta_{Knee}^{Des}$ because $K^{Syn}$, the sum of $K^{TO}$ and $\Delta K$, is equal to 0 for the duration of the phase. $K^{TO}$ sets the initial value of $K^{Syn}$ at TO and is equal to 0 in this stride because $\theta_{Knee}^{TO}$ is 43.55° (Fig. 4d). $\Delta K$ increments or decrements the value of $K^{Syn}$ throughout the phase and is set to a decrement in this experiment because $\theta_{Thigh}^{Meas}$ varies between 12° and -36° and $\dot{\theta}_{Thigh}^{\mathrm{Meas}}$ is always greater than -50deg/s (15). $\theta_{Knee}^{Meas}$ follows the desired $\theta_{Knee}^{MJ}$ position using PID control (Fig. S3b). This smoothly returns the knee from $\theta_{Knee}^{TO}$ equal to 48.2° and $\dot{\theta}_{Knee}^{TO}$ equal to 8.8 deg/s back to 0° and 0 deg/s by the end of the swing duration, $t^{swing}$ (13) (Fig. 4c). The unified *No Contact* controller enabled smooth walking swing and prepared the prosthesis for the next HS by utilizing the knee position at TO and the thigh kinematics throughout the phase to command a minimum jerk swing trajectory.

The ankle is controlled independently from the knee during both the *Contact* and *No Contact* states. In *Contact*, the ankle is controlled by the sum of virtual impedance torques $T_{Ankle}^{Damping}$ and $T_{Ankle}^{Stiffness}$ (6). $T_{Ankle}^{Damping}$ commands positive plantarflexion and negative dorsiflexion torque proportional to $\dot{\theta}_{Ankle}^{Meas}$ (15-16). Negative $T_{Ankle}^{Damping}$ is commanded near the beginning of *Contact* when the user is plantarflexing the foot to lie flat on the ground and again at the end of *Contact* when the foot plantarflexes to push off the ground (Fig. S3c). $T_{Ankle}^{Stiffness}$ is commanded throughout *Contact* as a function of the difference between $\theta_{Ankle}^{Meas}$ and the equilibrium angle, $\theta^{Eq},$ the sum of $\theta^{Eq1}$ and $\theta^{Eq2}$ (10-14) (Fig. 3a). In walking, $\theta^{Eq}$ is equal to only $\theta^{Eq1}$, the sum of $\theta_{Ankle}^{Meas}$ and $\theta_{Shank}^{Meas}$ (10). $\theta^{Eq2}$ is 0° throughout *Contact* due to near-zero $\theta_{Knee}^{Meas}$ at the beginning of *Contact* and greater than 10° $\theta_{Thigh}^{Meas}$ at the end of *Contact*, which sets the variable gain to $\theta^{Eq2}$, $K^{AK}$, equal to 0 (11) (Fig. 3c). Between 0-40% of *Contact*, $T_{Ankle}^{Stiffness}$ commands up to 10.9 Nm of plantarflexion torque due to a difference between $\theta_{Ankle}^{Meas}$ and $\theta^{Eq}$ of up to 3.1° (7) (Fig. S3c). $T_{Ankle}^{Stiffness}$ is added to up to -7.4 Nm of $T_{Ankle}^{Damping}$ during this period and results in total commanded torque that is largely net-zero (6). As the user rolls over their foot between 40% and 80% of contact, $T_{Ankle}^{Stiffness}$ commands up to 120 Nm of plantarflexion torque due to $\theta^{Eq1}$ increasing to its maximum value of 8° and creating a difference between $\theta_{Ankle}^{Meas}$ and $\theta^{Eq}$ of up to 13.8° (7) (Fig. S3c). $T_{Ankle}^{Stiffness}$ is added to near-zero $T_{Ankle}^{Damping}$ during this period (7). At 100% of *No Contact*, $\theta_{Ankle}^{Meas}$ increases to 4.8° and $T_{Ankle}^{Stiffness}$ decreases to 0 Nm (Fig. S3c). The ankle angle at TO, $\theta_{Ankle}^{TO}$, is used to calculate the duration of the knee’s minimum jerk trajectory, $t^{swing}$ (13) (Fig. 2c). During *No Contact*, the ankle is controlled with low PID gains to smoothly return to 0° and does not require a calculated swing trajectory. The unified controller enabled stable rollover, powered pushoff, and coordinated torque control with the knee by utilizing virtual impedance and the calculated equilibrium angle.


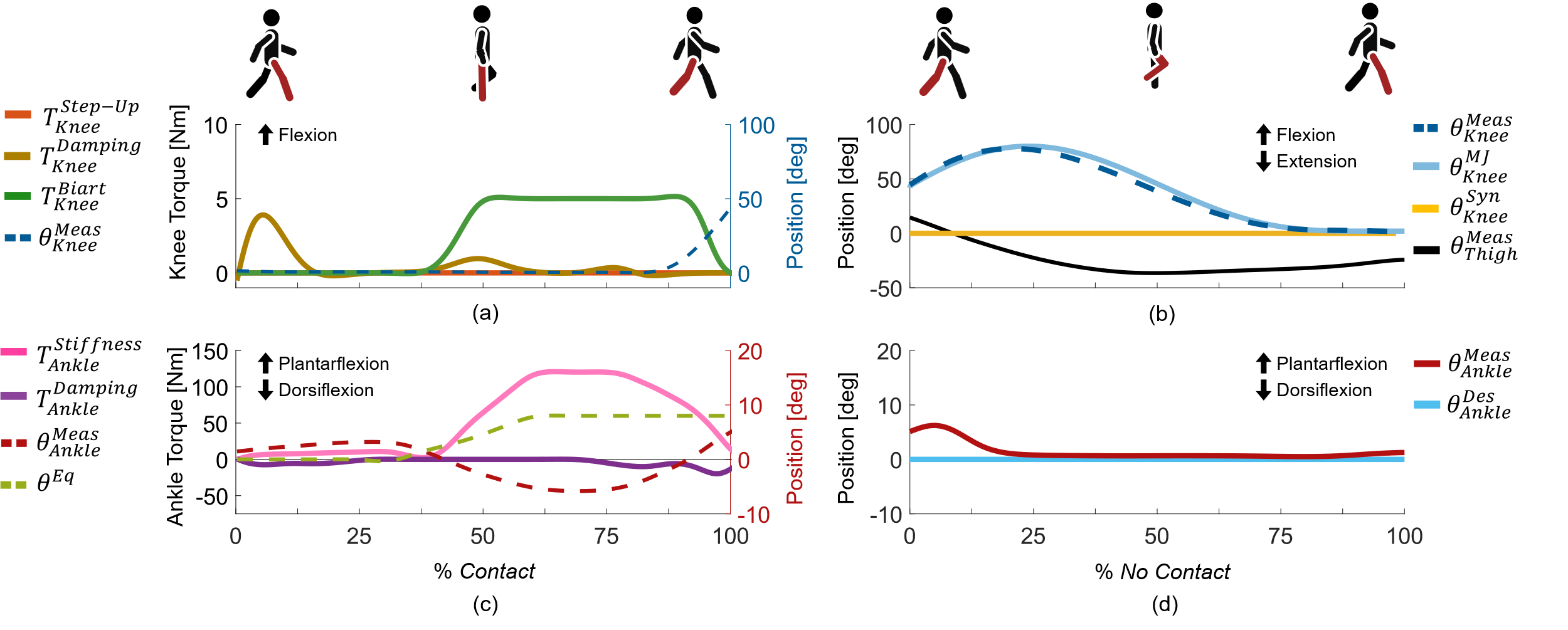


Fig. S3 Joint torque and positions from TF02 during a single level ground walking stride from HS to HS. (a) Commanded knee torques and knee position during *Contact.* (b) Measured knee position and desired positions during *No Contact*. (c) Commanded ankle torques and ankle position during *Contact.* (d) Measured ankle position and desired position during *No Contact*.

S5, Variable Speed Walking

The unified controller next demonstrated ankle damping adaptation to walking speed. Fig. S4 shows joint position, power, and control variables from TF03 walking on a level treadmill at three different speeds. As walking speed increased across the three tests, the subject’s horizontal hip velocity, $v_{Hip}^{Meas}$, also increased (Fig. S4a). $v_{Hip}^{Meas}$ is calculated continuously when the controller is in *Contact* using sensors onboard the prosthesis (S5). The average values of $v_{Hip}^{Meas}$ were 0.81 m/s, 1.05 m/s, and 1.27 m/s (Fig. S4a). As $v_{Hip}^{Meas}$ increased across the three tests, the commanded ankle damping coefficient, $B^{Ankle}$, decreased (Fig. S4b). The average values of $B^{Ankle}$ across the three tests were 1.14 Nm*s/deg, 0.77 Nm*s/deg, and 0.43 Nm*s/deg (Fig. S4b). Between 0-30% of *Contact,* $B^{Ankle}$ is 1.3 Nm*s/deg for all speeds because $\theta_{Shank}^{Meas}$ is less than 0° (S4) (Fig. S4b). Between 30-75% of *Contact*, the ankle dorsiflexes as the user rolls over the foot, which results in $B^{Ankle}$ equaling $B^{DF}$ (S4) (Fig. 3e). Between 75-100% of *Contact*, the ankle plantarflexes to push off the ground, which results in $B^{Ankle}$ equaling $B^{PF}$ (S4) (Fig. 3d). Both $B^{DF}$ and $B^{PF}$ decrease as $v_{Hip}^{Meas}$ increases (Fig. S4b). $B^{PF}$ decreases with higher sensitivity to $v_{Hip}^{Meas}$ compared to $B^{DF}$ (Fig. 3d,e) (Fig. S4b). The unified *Contact* controller demonstrated speed adaptation without extrinsic estimation or measurement of the user’s actual speed by modulating ankle damping as a function of $v_{Hip}^{Meas}$.

Modulating ankle damping simultaneously affected the behavior of other elements in the unified controller. Decreased ankle damping resulted in increased positive power as speed increased (Fig. S4c). The peak ankle power values were 44.8 W, 113.4 W, and 374.3 W, occurring at roughly 80% of *Contact* (Fig. S4c). The total energy provided from the prosthesis, calculated as the integral of power over time, increased across the three tests with values of -0.031 J/kg, 0.010 J/kg, and 0.015 J/kg. This change in power, calculated as the product of $\dot{\theta}_{Ankle}^{Meas}$ and $T_{Ankle}$, is a result of the decrease in $B^{Ankle}$ and the proportional decrease in $T_{Ankle}^{Damping}$ (S3). $\dot{\theta}_{Ankle}^{Meas}$ increased as $T_{Ankle}^{Damping}$ decreased because the user was able to roll over their foot faster. $T_{Ankle}$ increased as $T_{Ankle}^{Damping}$ decreased because $T_{Ankle}^{Stiffness}$ did not change, making the sum of $T_{Ankle}^{Stiffness}$and $T_{Ankle}^{Damping}$ increase during pushoff (6). This adaptation additionally resulted in a higher ankle angle at TO (Fig. S4d). As power increased across the three tests, the ankle angle at TO increased (Fig. S4d). $\theta_{Ankle}^{TO}$ was 1.2°, 2.5°, and 3.1° across the three speeds (Fig. S4d). As a result, the duration of the subsequent minimum jerk trajectory, $t^{swing}$, decreased proportionally to $\theta_{Ankle}^{TO}$ (13) (Fig. 4a-c). The unified controller increased power and energy injected into the stride and enabled speed adaptation during the subsequent *No Contact* control phase. These adaptations are achieved by modulating ankle damping as a function of $v_{Hip}^{Meas}.$


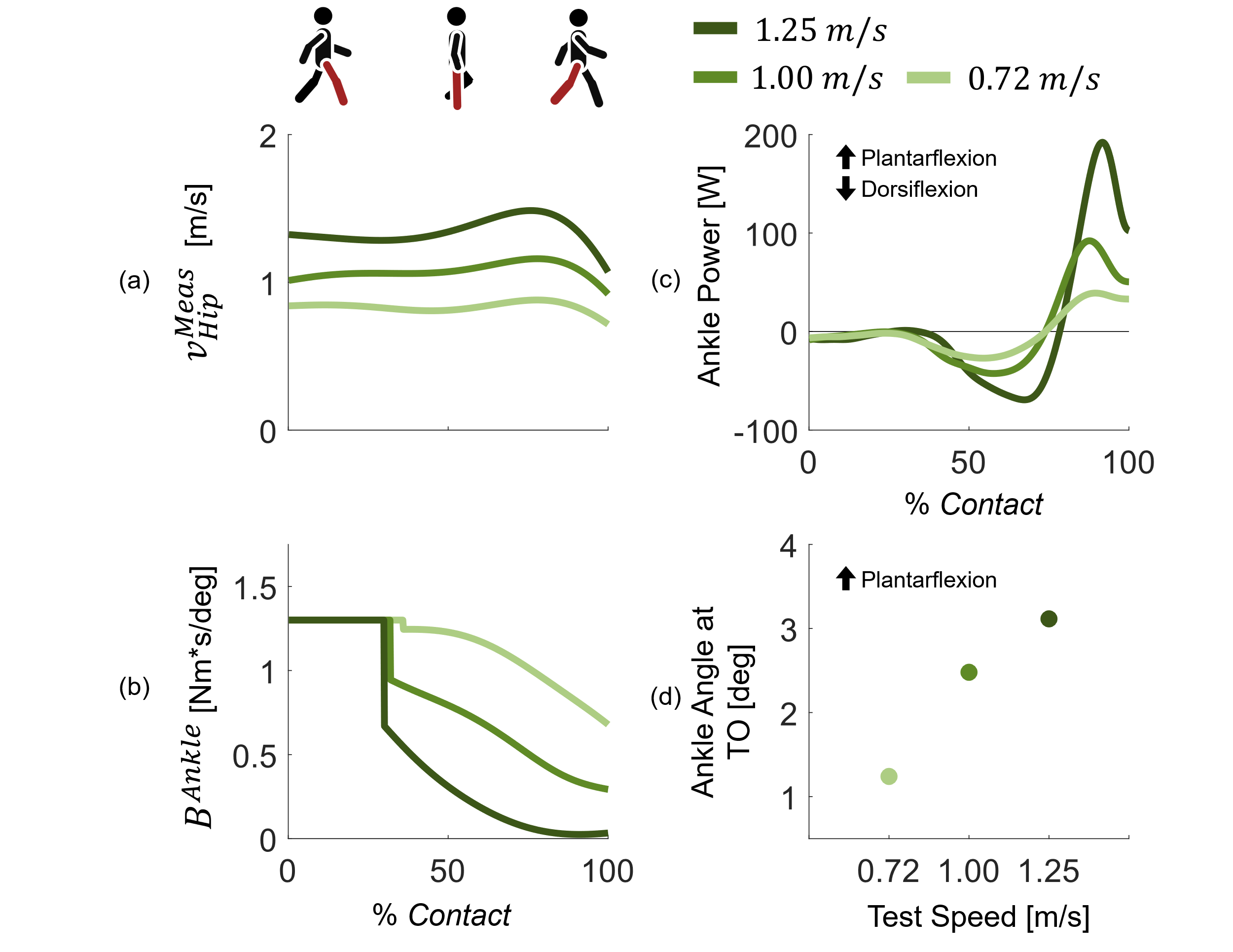


Fig. S4 Control variables during the *Contact* phase from TF01 during a single walking stride from HS to TO at three walking speeds. (a) Estimated horizontal hip velocity, $\boldsymbol{v}_{\boldsymbol{Hip}}^{\mathbf{Meas}}$. (b) Commanded ankle damping coefficient. (c) Ankle power. (d) Ankle angle at TO.

S5, Stair Ascent

The unified controller enabled controlled stance phase of stair ascent. Fig. S5 shows joint position and torque data from TF03 during stair ascent. The unified controller enters the *Contact* state at HS, or 0% of *Contact*. Between 0-60% of *Contact,* the knee extends from 60.8° ($\theta_{Knee}^{HS}$) to 0° (Fig. S5a). The bell-shaped $T_{Knee}^{Step-Up}$ torque curve is commanded, which peaks at 71.2 Nm of extension torque, based on the knee angle at HS (2-4) (Fig. 2a-b) (Fig. S5a). Between 30-60% of *Contact*, $T_{Knee}^{Damping}$ commands up to 26.9 Nm of flexion torque as $\theta_{Knee}^{Meas}$ decreases below 20°, which sets $B^{Ext}$ to its maximum value (7-8) (Fig. 2d). Between 60-100% of *Contact*, both $T_{Knee}^{Step-Up}$ and $T_{Knee}^{Damping}$ command near-zero torque (Fig. S5a). Between 70-90% of *Contact*, $T_{Knee}^{Biart}$ commands up to 4.2 Nm of flexion torque (Fig. S5a). This torque is proportional to $T_{Ankle}$ based on $K^{Biart}$, which both increase as the user leans forward on the step and the knee is fully extended against the end stop (5) (Fig. 2c) (Fig. S5a-b). Between 90-100% of *Contact*, $T_{Knee}^{Biart}$ decreases to 0 Nm (5) (Fig. 2c) (Fig. S5a). The unified *Contact* controller enabled controlled knee extension by utilizing the knee position at HS to define extension torque and position-dependent damping to define flexion torque.

The unified controller next enabled a smooth swing phase of stair ascent. The *No Contact* control state begins at TO, or 0% of *No Contact*. During this phase, $\theta_{Knee}^{MJ}$ is equal to 0 and $\theta_{Knee}^{Syn}$ dominates $\theta_{Knee}^{Des}$ because $K^{Syn}$, the sum of $K^{TO}$ and $\Delta K$, is equal to 1 (12). $K^{TO}$ sets the initial value of $K^{Syn}$ at TO and is equal to 1 because $\theta_{Knee}^{TO}$ is 7.2° (Fig. 4d). $\Delta K$ is positive for the entire phase because $\theta_{Thigh}^{Meas}$ decreases below -35° shortly after TO and $\dot{\theta}_{Thigh}^{\mathrm{Meas}}$ is less than -20°/s (15) (Fig. S5b). This increment does not change the value of $K^{Syn}$, as it has a maximum value of 1 (15). Between 0- 50% of *No Contact*, $\theta_{Knee}^{Syn}$ increases from 9.1° up to 67.4° (Fig. S5b). $\theta_{Knee}^{Syn}$ is calculated as the sum of three components, $\theta_{Knee}^{Pos}$, $\theta_{Knee}^{Vel}$, and $\theta_{Knee}^{Acc}$ based on the movement of the residual limb (20). Early in *No Contact*, $\theta_{Knee}^{Acc}$ increases up to 5.8° as the user lifts their body vertically using their contralateral limb and $\ddot{y}_{Thigh}^{Meas}$ increases (S9) (Fig. S2c) (Fig. S5b). $\theta_{\mathrm{Knee}}^{Vel}$ also increases up to 23.3° as the user quickly flexes their thigh and $\dot{\theta}_{Thigh}^{Meas}$ decreases (S8) (Fig. S2b) (Fig. S5b). $\theta_{Knee}^{Pos}$ increases gradually between 0-50% as a function of $\theta_{Thigh}^{Meas}$, plateauing at 62.9° as the user holds their thigh flexed above the next step (S7) (Fig. S5b). The unified *No Contact* controller enabled smooth stair ascent swing by utilizing the kinematics of the prosthesis and the user’s residual thigh to command a synergy-based desired position.

Ankle control during stance supported stable stair ascent. Total $T_{Ankle}$ during *Contact* is calculated as the sum of $T_{Ankle}^{Stiffness}$ and $T_{Ankle}^{Damping}$ (6). Between 0-45% of *Contact,* these torques cumulatively command between 0.2 and 12.0 Nm of plantarflexion torque (Fig. S5c). $T_{Ankle}^{Stiffness}$ is commanded as a function of the difference between $\theta_{Ankle}^{Meas}$ and $\theta^{Eq}$, which is the sum of $\theta^{Eq1}$ and $\theta^{Eq2}$ (10,12). $\theta^{Eq1}$ is near-zero between 0-45% due to $\theta_{Ankle}^{Meas}$ and $\theta_{Shank}^{Meas}$ being roughly equal magnitude and opposite sign (10). $\theta^{Eq}$ is thus dominated by $\theta^{Eq2}$, which is the product of the knee-ankle synergy angle, $\theta^{AK}$, and its variable gain, $K^{AK}$ (11). At 0% of *Contact*, $\theta^{AK}$ is -12.0° and $K^{AK}$ is 1, due to $\theta_{Knee}^{Meas}$ being 60.8° and $\theta_{Thigh}^{Meas}$ -49.2° (11) (Fig. 3b-c) (Fig. S5c). Between 0-45% of *Contact*, $\theta^{Eq2}$ increases from -12.0° to -1.0° as $\theta_{Knee}^{Meas}$ decreases (Fig. S5a,c). $T_{Ankle}^{Damping}$ of up to 22.3 Nm in dorsiflexion torque is commanded as the ankle plantarflexes (S3) (Fig. S5c). Between 50-90% of *Contact*, $T_{Ankle}^{Stiffness}$ and $T_{Ankle}^{Damping}$ increase and reach a cumulative peak of 31.3 Nm of plantarflexion torque as the user leans forward to place their contralateral limb on the next stair (Fig. S5c). Between 90-100% of *Contact*, $\theta^{Eq1}$ increases as the user leans further forward and the sum of $\theta_{Ankle}^{Meas}$ and $\theta_{Shank}^{Meas}$ increases while $\theta^{Eq2}$ is zero (12,13). The unified *Contact* controller enabled appropriate ankle function during stair ascent stance by utilizing the synergy-based $\theta^{Eq2}$ to plantarflex the ankle and keep the foot flat on the step.

Ankle control during swing supported smooth stair ascent. During the *No Contact* phase, the ankle follows $\theta_{Ankle}^{Des}$, calculated as the product of $\theta_{Ankle}^{Syn}$, a synergy-based angle, and $K^{Syn}$, the same variable gain utilized for control of the knee (18,26). $\theta_{Ankle}^{Syn}$ dominates $\theta_{Ankle}^{Des}$ because $K^{Syn}$ is equal to 1, as calculated for the knee (24-26). Between 0-25% of *No Contact*, $\theta_{Ankle}^{Syn}$ increases to 2.6° before decreasing to -14.0° between 25-100% (Fig. S5d). $\theta_{Ankle}^{Syn}$ is the sum of two components, $\theta_{Ankle}^{Pos}$ and $\theta_{Ankle}^{Acc}$, which are determined based on $\theta_{Knee}^{Meas}$ and the movement of the residual limb (S10). Between 0-25% of *No Contact*, $\theta_{\mathrm{Ankle}}^{Acc}$ increases to 9.2° as the user lifts their body vertically using their contralateral limb and $\ddot{y}_{Thigh}^{Meas}$ increases (S12) (Fig. S2c) (Fig. S5d). Between 0-50% of *No Contact*, $\theta_{Ankle}^{Pos}$ decreases linearly as a function of $\theta_{Knee}^{Meas}$, plateauing at -14.9° (S11) (Fig. S5d). This dorsiflexion keeps the foot parallel to the ground and allows it to land flat on the next stair. The unified *No Contact* controller enabled appropriate foot placement by utilizing $K^{Syn}$ to coordinate synergy-based position control in the knee and ankle simultaneously.


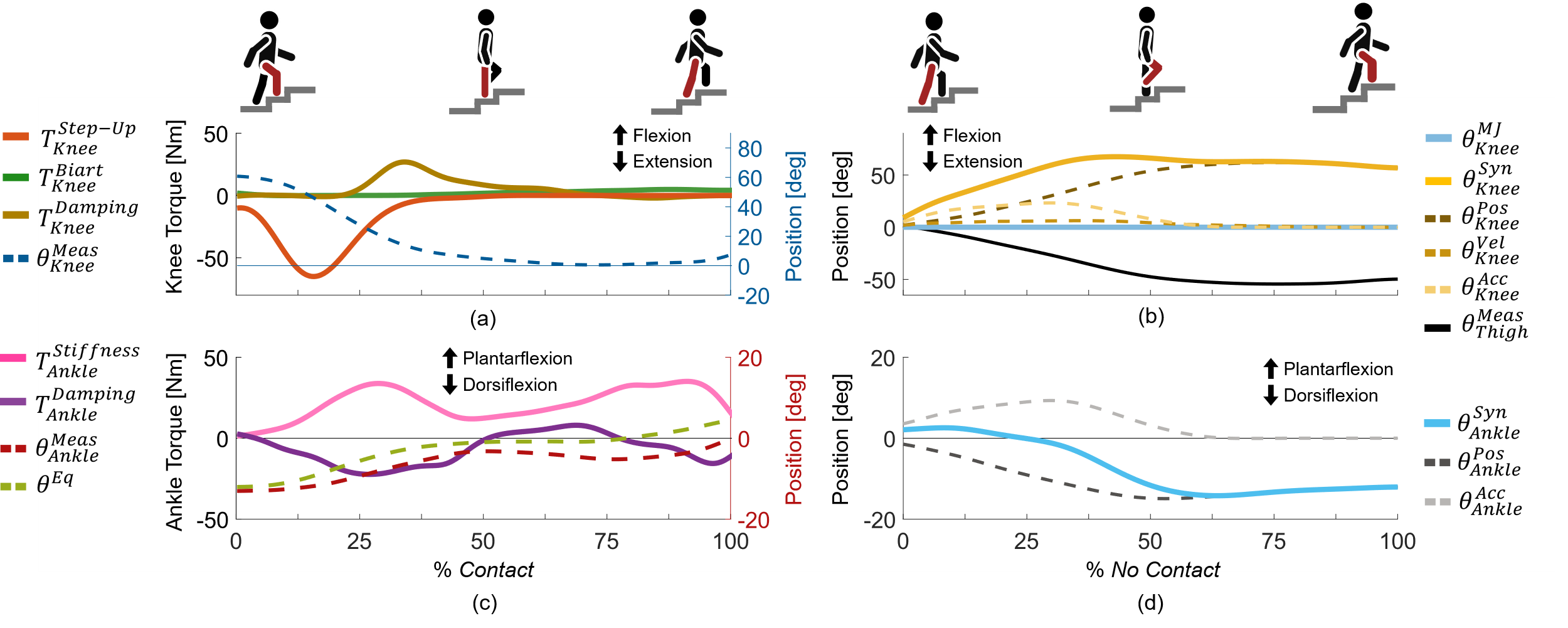


Fig. S5 Joint torque and positions from TF03 during a single stair ascent stride from HS to HS. (a) Commanded knee torques and knee position during *Contact.* (b) Measured knee position and desired positions during *No Contact*. (c) Commanded ankle torques and ankle position during *Contact.* (d) Measured ankle position and desired position during *No Contact*.

S6, Rough Terrain

The unified controller enabled continuous ankle adaptation to variable, uneven terrain. Joint position and torque data from TF02 taking three consecutive steps on incline, level, and decline terrain, respectively, is shown in Fig. S6. In the first *Contact*, between 0-1.1 s, the prosthesis steps on a 10% incline block and $\theta_{Ankle}^{HS}$ is 0° (Fig. S6a). The ankle then dorsiflexes to -16.2° during rollover and plantarflexes to 2.3° during pushoff (Fig. S6a). $\theta_{Shank}^{HS}$ is -20.7° and increases to 56.2° at TO (Fig. S6a). $\theta^{Eq}$, the sum of $\theta^{Eq1}$ and $\theta^{Eq2}$, is 0° until $\theta_{Shank}^{Meas}$ increases above -5° at around 0.3 s (Fig. 3a). $\theta^{Eq2}$ is 0° throughout the phase because the knee is extended until just before TO, at which point $\theta_{Thigh}^{Meas}$ is greater than 10° and $K^{AK}$ is set to 0 (1) (Fig. 3c). $\theta^{Eq1}$ therefore dominates $\theta^{Eq}$. Between 0.3-0.7 s, $\theta^{Eq1}$ decreases to -7.9° because its components, $\theta_{Ankle}^{Meas}$ and $\theta_{Shank}^{Meas}$, are negative and near-zero, respectively, during this period (12,13) (Fig. S6a). Between 0.7-1.1 s, $\theta^{Eq1}$ increases up to its maximum of 8° as $\theta_{Shank}^{Meas}$ becomes much greater than $\theta_{Ankle}^{Meas}$ (Fig. S6a). The difference between the calculated $\theta^{Eq}$ and $\theta_{Ankle}^{Meas}$ is used to determine $T_{Ankle}^{Stiffness}$ (7). During *Contact*, $T_{Ankle}^{Stiffness}$ commands up to 120 Nm of plantarflexion torque as the difference between $\theta^{Eq}$ and $\theta_{Ankle}^{Meas}$ increases up to 22.8° (7) (Fig. S6a,b). $T_{Ankle}^{Damping}$ commands up to 36.9 Nm of plantarflexion torque during rollover and 54.2 Nm of dorsiflexion torque during pushoff (Fig. S6b).

In the second *Contact*, between 1.7-2.2 s, the prothesis steps on level ground. The ankle plantarflexes to 3.5° to reach foot flat, dorsiflexes to -4.6° during rollover, then plantarflexes to 2.7° at TO (Fig. S6a). $\theta_{Shank}^{Meas}$ follows a similar trajectory to the previous stride. $\theta^{Eq1}$ is near-zero during rollover in this stride because the ankle does not dorsiflex significantly compared to the previous stride (Fig. S6a). $T_{Ankle}^{Stiffness}$ commands up to only 111.9 Nm of plantarflexion torque on this stride, as the difference between $\theta^{Eq}$ and $\theta_{Ankle}^{Meas}$ reaches 12.5° (Fig. S6a,b). $T_{Ankle}^{Damping}$ is also reduced during this stride, commanding 20.1 Nm of plantarflexion torque during rollover and 22.9 Nm of flexion torque during pushoff due to the decreased ankle movement (Fig. S6b).

In the third *Contact* state, between 3.6-4.6 s, the prothesis steps on a 10° decline block. The ankle plantarflexes up to 6.6° to reach foot flat, dorsiflexes to 1.1°, then plantarflexes to 4.4° (Fig. S6a). $\theta_{Shank}^{Meas}$ follows a similar trajectory to the previous strides. $\theta^{Eq1}$ reaches its maximum of 8° during rollover of this stride due to the positive $\theta_{Ankle}^{Meas}$ and remains at 8° for the rest of the state (Fig. S6a). $T_{Ankle}^{Stiffness}$ commands just 61.0 Nm of plantarflexion torque as a result of the difference between $\theta^{Eq}$ and $\theta_{Ankle}^{Meas}$ reaching only 6.8°, which allows the ankle to plantarflex and remain flat on the ground throughout the state. The unified controller enabled continuous adaptation to terrain on three consecutive, highly variable steps by utilizing the calculated equilibrium angle, $\theta^{Eq1}$ to modulate ankle impedance.


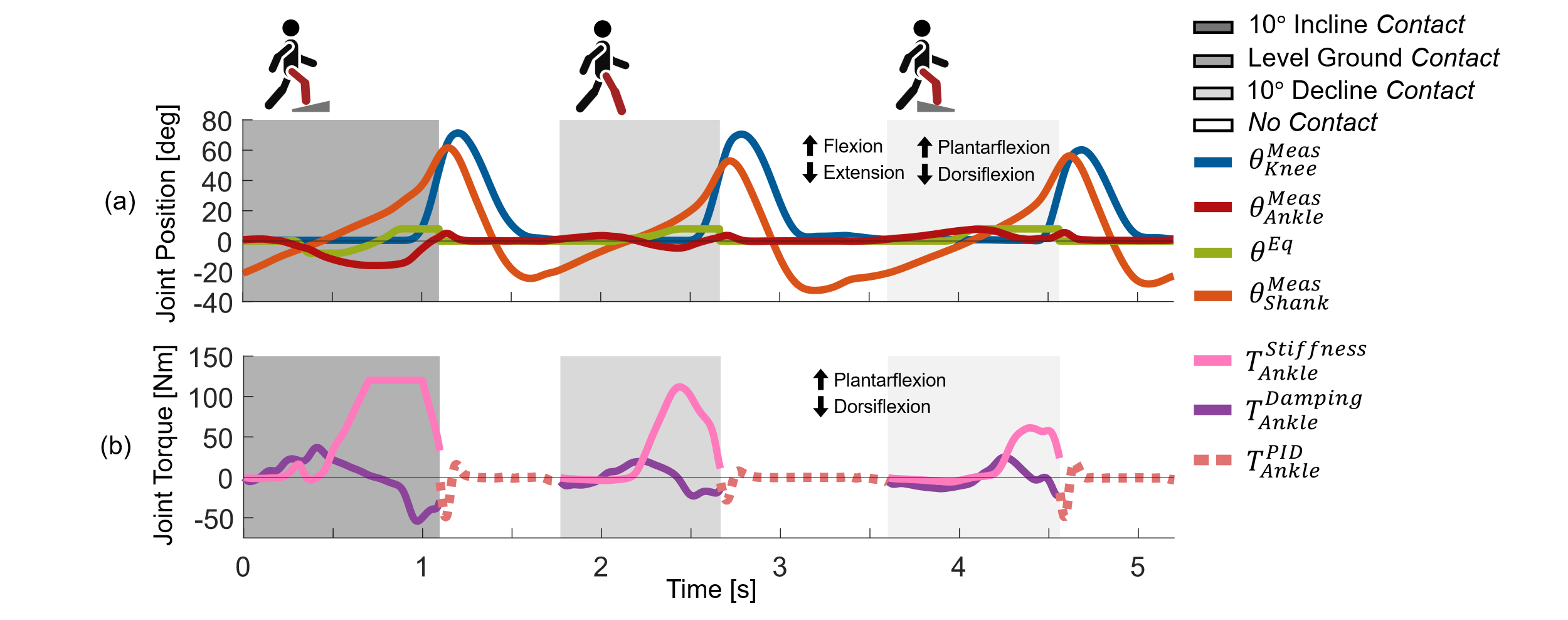


Fig. S6 Joint position and torque from TF02 taking three steps on incline, level, and decline terrain, respectively. (a) Measured knee, ankle, and shank positions and calculated ankle equilibrium angle. (b) Commanded ankle torques.

S7, Sit to Stand

The unified controller enabled a stand-to-sit transition. Joint position, torque, and power data from TF01 performing a continuous stand-to-sit-to-stand transition is shown in Fig. S7. The subject begins the stand-to-sit transition at 0 s. The unified controller is in the *Contact* state as the user’s sound limb and prosthesis are weighted evenly. Between 0-2 s, the user sits and $\theta_{Knee}^{Meas}$ increases from 0° to 110° (Fig. S7a). $T_{Knee}^{Damping}$ commands extension torque of up to 29.0 Nm proportional to $\dot{\theta}_{Knee}^{\mathrm{Meas}}$ based on $B^{Flex}$ (Fig. S7b). $B^{Flex}$ is equal to its maximum value throughout the stand-to-sit movement because $\theta_{Thigh}^{Meas}$ is always less than 5° (7,8) (Fig. 2e) (Fig. S7a). Between 0-2 s, $\theta_{Ankle}^{Meas}$ decreases from 0° to -18° (Fig. S7a). $\theta^{Eq}$, the sum of $\theta^{Eq1}$ and $\theta^{Eq2}$, decreases from 0° to -22° (9) (Fig. S7a). $\theta^{Eq2}$ dominates $\theta^{Eq}$ during the movement due to the knee-ankle synergy angle, $\theta^{AK}$, increasing proportional to $\theta_{Knee}^{Meas}$ (11). $T_{Ankle}^{Stiffness}$, determined as a function of the difference between $\theta_{Ankle}^{Meas}$and $\theta^{Eq}$, commands up to 18.6 Nm of plantarflexion torque during the movement (7) (Fig. S7c). $T_{Ankle}^{Damping}$ commands up to 17.2 Nm of plantarflexion torque as a function of $\dot{\theta}_{Ankle}^{Meas}$ (Fig. S7c). During the transition, the knee and ankle produce net negative power and the prosthesis absorbs 0.51 J/kg of energy (Fig. S7d). The unified controller enabled a slow and stable stand-to-sit transition by utilizing virtual impedance control in the knee and ankle joints.

The unified controller enabled a sit-to-stand transition. Before beginning the sit-to-stand transition, between 2-2.5 s, the prosthesis is unweighted and the unified controller briefly enters the *No Contact* state. However no movement is commanded (Fig. S7a). $\theta_{Knee}^{Meas}$ does not change because $K^{Syn}$ is equal to 1 during this period, causing $\theta_{Knee}^{Syn}$ to dominate $\theta_{Knee}^{Des}$ and set a high desired knee angle proportional to $\theta_{Thigh}^{Meas}$ (18,24) (Fig. S2a) (Fig. S7a). The user begins the stand-to-sit transition at 2.5 s. The unified controller re-enters the *Contact* state as the user weights the prosthesis. Between 2.5 s and 4 s, the user stands and $\theta_{Knee}^{Meas}$ decreases from 110° to 0° (Fig. S7a). The bell-shaped $T_{Knee}^{Step-Up}$ torque curve is commanded, which peaks at 72.8 Nm of extension torque, based on $\theta_{Knee}^{HS}$ being equal to 110° (2-4) (Fig. 1a,b) (Fig. S7b). $T_{Knee}^{Damping}$ commands up to 34.8 Nm of flexion torque, slowing down the movement near full extension (Fig. S7b). This torque results from $\theta_{Knee}^{Meas}$ decreasing below 20°, setting $B^{Ext}$ to its maximum value (7-8) (Fig. 2d) (Fig. S7a). In the ankle, $T_{Ankle}^{Stiffness}$ commands up to 44.7 Nm of plantarflexion torque as $\theta^{Eq}$ increases from -22° to 0° (12,14) (Fig. 3b) (Fig. S7c). Between 2.5 s and 3 s, the sum of positive $T_{Ankle}^{Stiffness}$ and near-zero $T_{Ankle}^{Damping}$ is positive, resulting in plantarflexion of the ankle as $\theta_{Knee}^{Meas}$ decreases (Fig. S7c). Between 3-4 s, positive $T_{Ankle}^{Stiffness}$ and negative $T_{Ankle}^{Damping}$ summate to a largely net-zero commanded $T_{Ankle}$, allowing the ankle to plantarflex as the knee extends (Fig. S7c). During this movement, the sum of positive knee power and negative ankle power is net positive, and the prosthesis injects 1.14 J/kg of energy (Fig. S7d). The unified controller enabled controlled sit-to-stand by utilizing biomechanical markers such as $\theta_{Knee}^{HS}$ to command the same torque and impedance controls seen in stair ascent, enabling both activities without mode selection or activity classification.


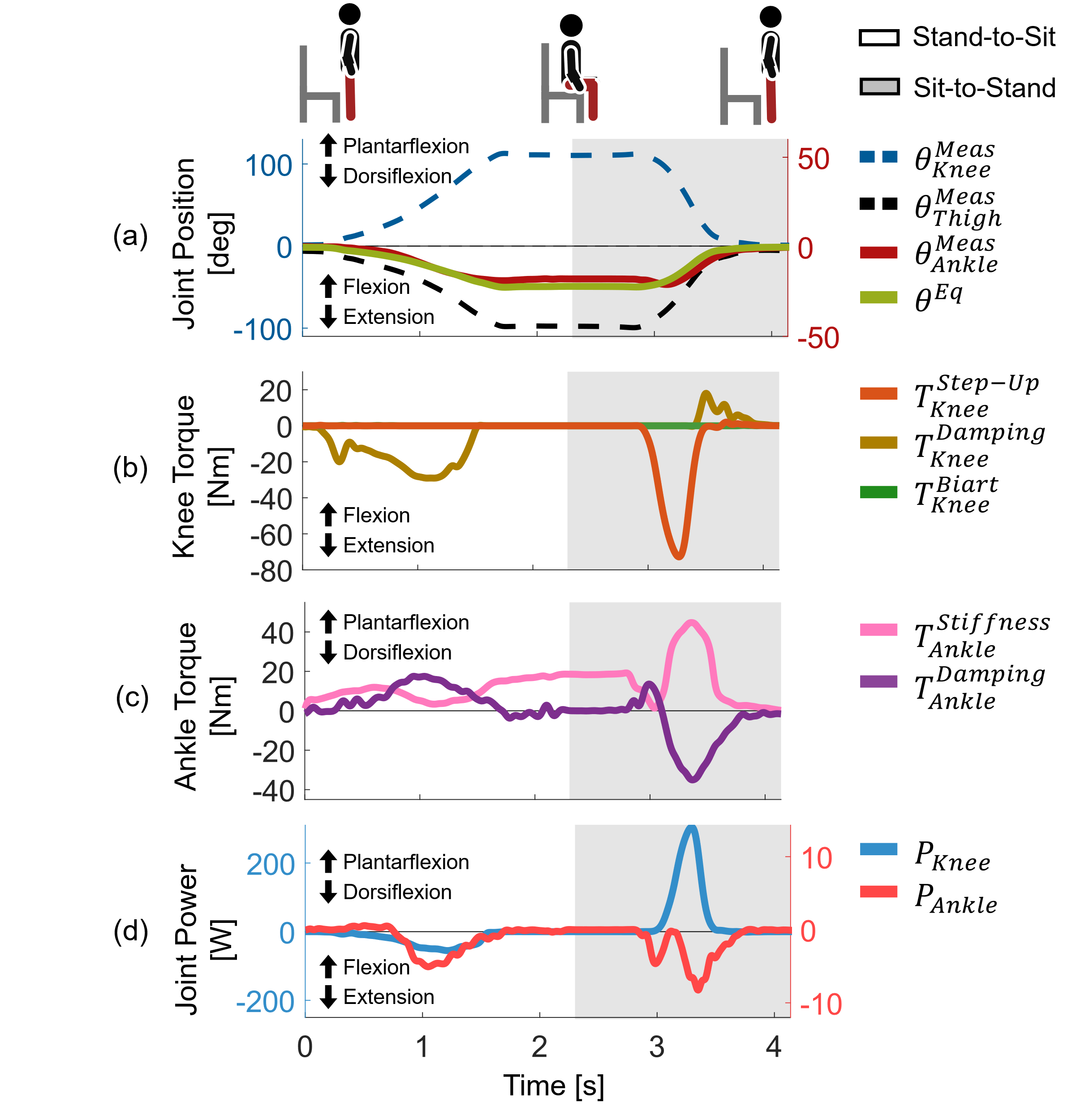


Fig. S7 Joint position, torque, and power from TF01 during a sit-to-stand and stand-to-sit transition. (a) Joint and body segment positions. (b) Commanded knee torques. (c) Commanded ankle torque. (d) Joint power calculated as $\boldsymbol{T*}{\dot{\boldsymbol{\theta}}}^{\boldsymbol{Meas}}$.

S8, Walk-Stair Ascent-Walk Transitions

To demonstrate the controller’s ability to transition between ambulation activities, the subjects performed four activities that consisted of walking, transitioning to either stair ascent (SA) or stair descent (SD), and transitioning back to walking. Subjects also alternated leading the first stair with their sound side first (SSF) and with their prosthesis side first (PSF), making eight total transitions demonstrated in the four experiments shown below. For each subject, we collected three trials and averaged the data to demonstrate the functionality of the controller.

*S8i, Walk-Stair Ascent-Walk PSF*

The unified controller enabled transitions between walking and stair ascent, leading with the prosthesis. Fig. S8i shows joint position and torque data from TF01 during a four-step sequence in which they transition from walking to stair ascent and back to walking. The first stride is a level ground walking stride starting from standing. During the second stride, the user transitions from walking to stair ascent, leading with the prosthesis, resulting in an activity transition over the course of the *No Contact* state. The third stride is a stair ascent stride equivalent to steady state stair ascent (Fig. S5). During the fourth stride, the user transitions from stair ascent back to walking after climbing the last step with their sound limb, requiring the prosthesis to clear the last step before transitioning back to walking. The first and third strides of the sequence are identical to the steady state activities shown in Fig. S3 and Fig. S5. However, the second and fourth strides require different biomechanics during *No Contact* to transition between activities. The unified controller is able to generate the required biomechanics to enable transitions between walking and stair ascent.

The unified controller enabled a walking to stair ascent transition during *No Contact* of the second stride of the sequence. During *Contact*, beginning at 1.5 s, the kinematics of the user’s residual limb and prosthesis are equivalent to level ground walking, and the unified controller therefore commands the same control efforts seen in steady state walking (Fig. S3). During *No Contact*, the user transitions to stair ascent leading with their prosthesis. In order to clear the ground, the knee must be flexed at TO. Moreover, in order to clear the first step, the knee must remain flexed up until HS instead of extending as needed for level ground walking. $\theta_{Knee}^{Des}$ is initially dominated by $\theta_{Knee}^{MJ}$ because $\theta_{Knee}^{TO}$ is 41.3°, which sets $K^{TO}$, the initial value of $K^{Syn}$, to 0 (Fig. S9a). However, over the first half of *No Contact*, $K^{Syn}$ increases from 0 to 1 and $\theta_{Knee}^{Des}$ transitions from being dominated by $\theta_{Knee}^{MJ}$ to being dominated by $\theta_{Knee}^{Syn}$ (12) (Fig. S8i). The increase in $K^{Syn}$ is the result of its calculation as the sum of $K^{TO}$ and a positive or negative constant, $\Delta K$ (14). Due to the movement of the user’s residual limb, $\Delta K$ is set to a positive constant, increasing $K^{Syn}$ (15). Specifically, $\Delta K$ is positive because $\theta_{Thigh}^{Meas}$ is less than -35°, $\dot{\theta}_{Thigh}^{Meas}$ is less than -20°/s, and $\theta_{Knee}^{Meas}$ is greater than 20° (15) (Fig. S8i). At the end of *No Contact,* the unified controller is commanding torque and desired positions equivalent to steady-state stair ascent, and the user has fully transitioned between activities. By continuously modulating $K^{Syn}$ from 0 to 1 the unified controller enabled walking to stair ascent transition within a single *No Contact* state.

The unified controller enabled a stair ascent to walking transition during *No Contact* of the fourth stride of the sequence. During *Contact*, beginning at 5.75 s, the kinematics of the user’s residual limb and prosthesis are equivalent to stair ascent, and the unified controller therefore commands the same control efforts seen in steady state walking (Fig. S3). During *No Contact*, the user transitions from stair ascent back to level ground walking, requiring the knee to flex after TO to clear the final step, then extend to land on level ground. $\theta_{Knee}^{Des}$ is initially dominated by $\theta_{Knee}^{Syn}$ because $\theta_{Knee}^{TO}$ is 2.5°, which sets $K^{TO}$ to 1 (Fig. 3d) (Fig. S8i). Due to the movement of the user’s residual limb, $\Delta K$ is set to a negative constant, decreasing $K^{Syn}$ (15). Specifically, $\Delta K$ is negative because $\theta_{Thigh}^{Meas}$ is between -10° and -35° and $\dot{\theta}_{Thigh}^{Meas}$ is greater than -50°/s (15) (Fig. S8i). As $K^{Syn}$ decreases from 1 to 0, $\theta_{Knee}^{Des}$ transitions from being dominated by $\theta_{Knee}^{Syn}$ to being dominated by $\theta_{Knee}^{MJ}$ (12) (Fig. S8i). By continuously modulating $K^{Syn}$ from 1 to 0 based on the movement of the user’s residual limb, the unified controller enabled a walking to stair ascent transition within a single *No Contact* state.

*S8ii, Walk-Stair Ascent-Walk SSF*

Fig. S8ii shows joint position and torque data from TF03 during a four-step sequence in which they transition from walking to stair ascent and back to walking. The first stride is a level ground walking stride starting from standing. During the second stride, the user transitions from walking to stair ascent, leading with their sound limb on the first step. The third stride is a stair ascent stride equivalent to steady state stair ascent (Fig. S5). During the fourth stride, the user transitions from stair ascent back to walking, climbing the last step with the prosthesis and then beginning level ground walking. The first and third strides of the sequence are identical to the steady state activities shown in Fig. S3 and Fig. S5. However, the second and fourth strides are transition strides that require unique control functionality. The unified controller enabled both steady-state activities and transition strides.


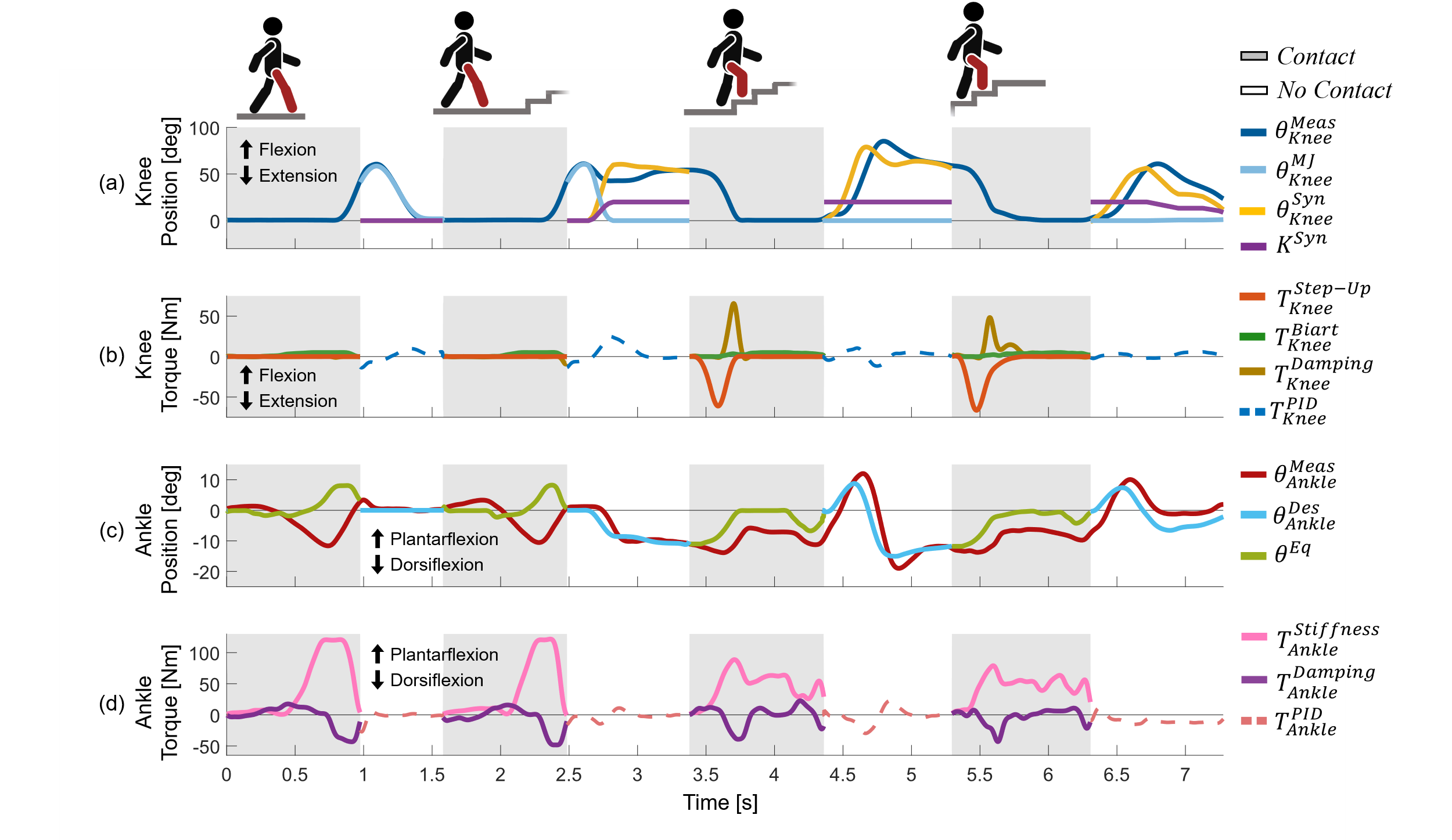


Fig. S8i *Walk-Stair Ascent-Walk PSF.* (a) Knee positions, (b) knee torques, (c) ankle positions, and (d) ankle torques from TF01 during the four-stride Walk-Stair Ascent-Walk sequence leading with the prosthesis.

The unified controller enabled walking to stair ascent transitions during the second stride of the sequence. In the first half of *Contact*, beginning at 1.5 s, the kinematics at HS and during rollover are equivalent to level ground walking, and the unified controller therefore commands the same control efforts seen in steady state walking (Fig. S3). At the same time, the user lifts their sound limb and places it on the first step. As the sound limb extends, the prosthesis is lifted, and the unified controller commands a desired position to climb the second step. During this *No Contact*, $\theta_{Knee}^{Syn}$ dominates $\theta_{Knee}^{Des}$ because $\theta_{Knee}^{TO}$ is 8.1°, and the user lifts their residual limb quickly, which together set $K^{Syn}$ to 1 (18,20,24) (Fig. S8ii). The knee flexes up to 57.9° to clear the step before the next $\theta_{Knee}^{HS}$. Due to high flexion at HS, the unified controller commands $T_{Knee}^{Step-Up}$ to lift the user up the step, equivalent to steady state stair ascent (Fig. S5) (Fig. S8ii). By defining $\theta_{Knee}^{Des}$ based on the knee angle at TO, the unified controller enabled walking to stair ascent transitions.

The unified controller enabled a stair ascent to walking transition during the fourth stride of the sequence. In the first half of *Contact*, beginning at 6 s, the kinematics at HS are equivalent to stair ascent, and the unified controller therefore commands the same control efforts seen in steady state stair ascent (Fig. S5). As the prosthesis extends, the users sound limb flexes to clear the final step and extends to make contact with level ground and begin walking. This causes the user to lean forward as they lengthen their stride. Leaning forward increases ankle dorsiflexion and results in high $T_{Ankle}^{Stiffness}$, which subsequently increases $T_{Knee}^{Biart}$ and causes the knee to flex up to 42.8° at TO (7) (Fig. S8ii a,b,d). In the final *No Contact*, $\theta_{Knee}^{MJ}$ dominates $\theta_{Ankle}^{Des}$ because $\theta_{Knee}^{TO}$ is 42.8° and the user swings their residual limb slowly, which together set $K^{Syn}$ to 0 (12) (Fig. S8ii). The knee is fully extended before the next HS, equivalent to steady state walking. By defining $\theta_{Knee}^{Des}$ based on the knee angle at TO, which is higher in the stair ascent to walking transition than in the walking to stair ascent transition.


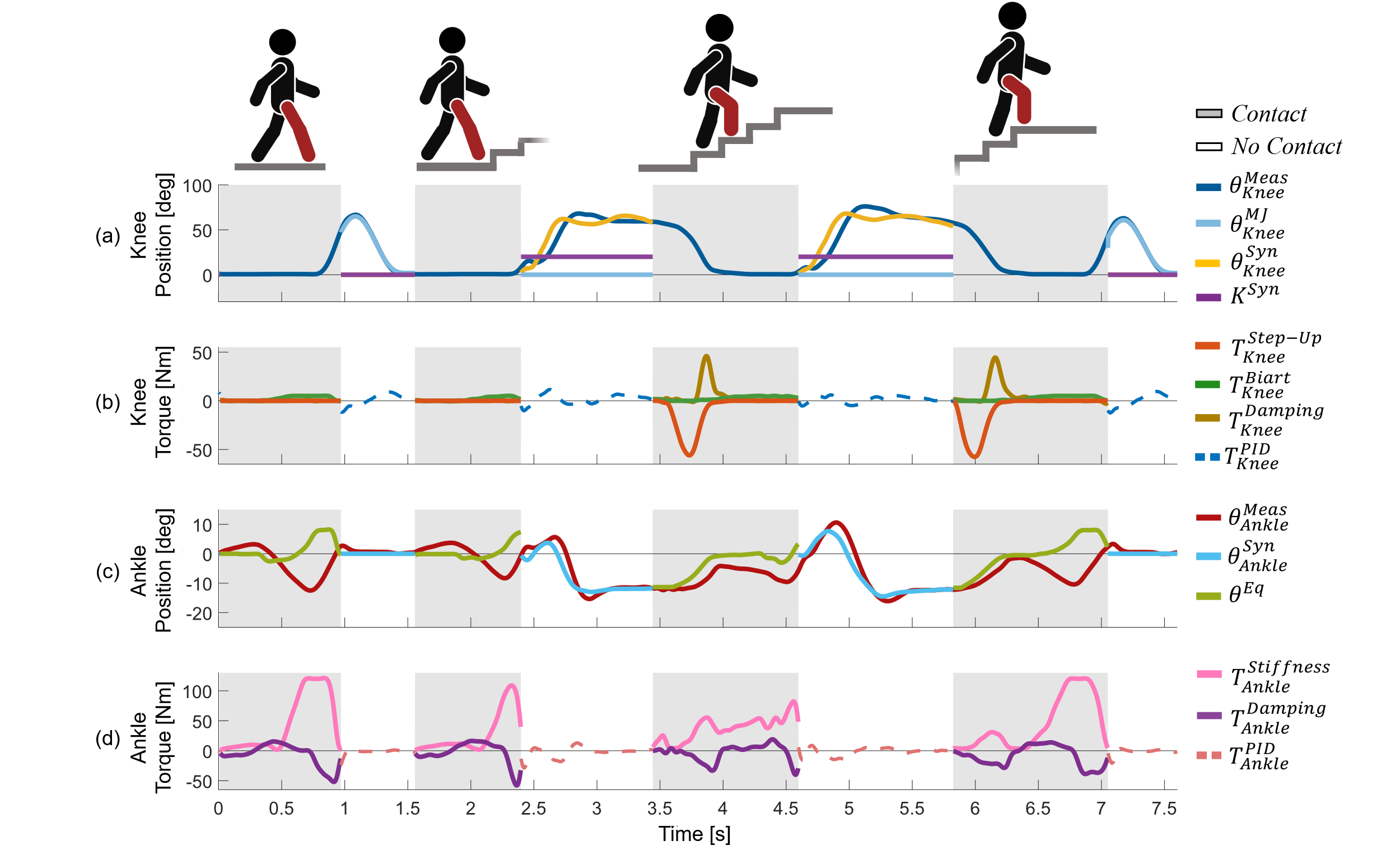


Fig. 8ii *Walk-Stair Ascent-Walk SSF.* (a) Knee positions, (b) knee torques, (c) ankle positions, and (d) ankle torques from TF03 during the four-stride Walk-Stair Ascent-Walk sequence leading with the subject’s sound limb.

*S8iii, Walk-Stair Descent-Walk PSF*

The unified controller enabled transitions between walking and stair descent, leading with the prosthesis. Fig. S8iii shows joint positions from TF02 during a four-step sequence transitioning from walking to stair descent and back to walking leading with the prosthesis. The first stride in the sequence is a walking stride starting from standing. During the second stride, the user transitions from walking to descending a set of stairs, leading with the prosthesis. The third stride is a stair descent stride, requiring the unified controller to provide high damping and smoothly flex the knee as the user descends. During the fourth stride, the user transitions from stair descent back to walking. The second and fourth strides of the sequence are transitions, requiring unique functionality from the unified controller. The unified controller enabled stair descent and transitions between stair descent and walking in a continuous sequence.

In the second stride, beginning at 1.75 s, $\theta_{Knee}^{HS}$ is 0.9° as the prosthesis lands on the edge of the stairs (Fig. S8iii). The knee then flexes up to 81.7° as the user descends the first step and places their sound limb on the next step (Fig. S10a). While in this *Contact* state, $T_{Knee}^{Step-Up}$ commands 0 Nm of torque because $\theta_{Knee}^{HS}$ is less than $\theta_{Knee}^{End}$ (2-4) (Fig. 2a,b) (Fig. S6a,b). At the same time, $T_{Knee}^{Damping}$ commands up to 47.9 Nm of extension torque because $B^{Flex}$ is at its maximum due to $\theta_{Thigh}^{Meas}$ being less than 5° throughout the movement (Fig. 2e) (Fig. S6b). In the ankle, although $\theta^{Eq}$ changes, $T_{Ankle}^{Stiffness}$ and $T_{Ankle}^{Damping}$ command near-zero torque because $K^{Stiffness}$ is 1 when $\theta_{Ankle}^{Meas}$ is greater than $\theta^{Eq}$ (11) (Fig. S8iii). The kinematics of the prosthesis during the third stride, occurring between 3.5-4.75 s, are identical to the second stride (Fig. S6). In the fourth stride, beginning at 4.75 s, the prosthesis lands on level ground and the user swings their sound limb forward starting from the final step. $\theta_{Knee}^{HS}$ is 5.9° and the knee extends to 0° as the user weights the prosthesis (Fig. S8iii). The prosthesis kinematics are equivalent to level ground walking, and the unified controller therefore commands the same control elements as seen in steady state walking (Fig. S3). By using impedance torque as a function of the position of the user’s residual limb the unified controller enabled transitions between walking and stair descent leading with the prosthesis.


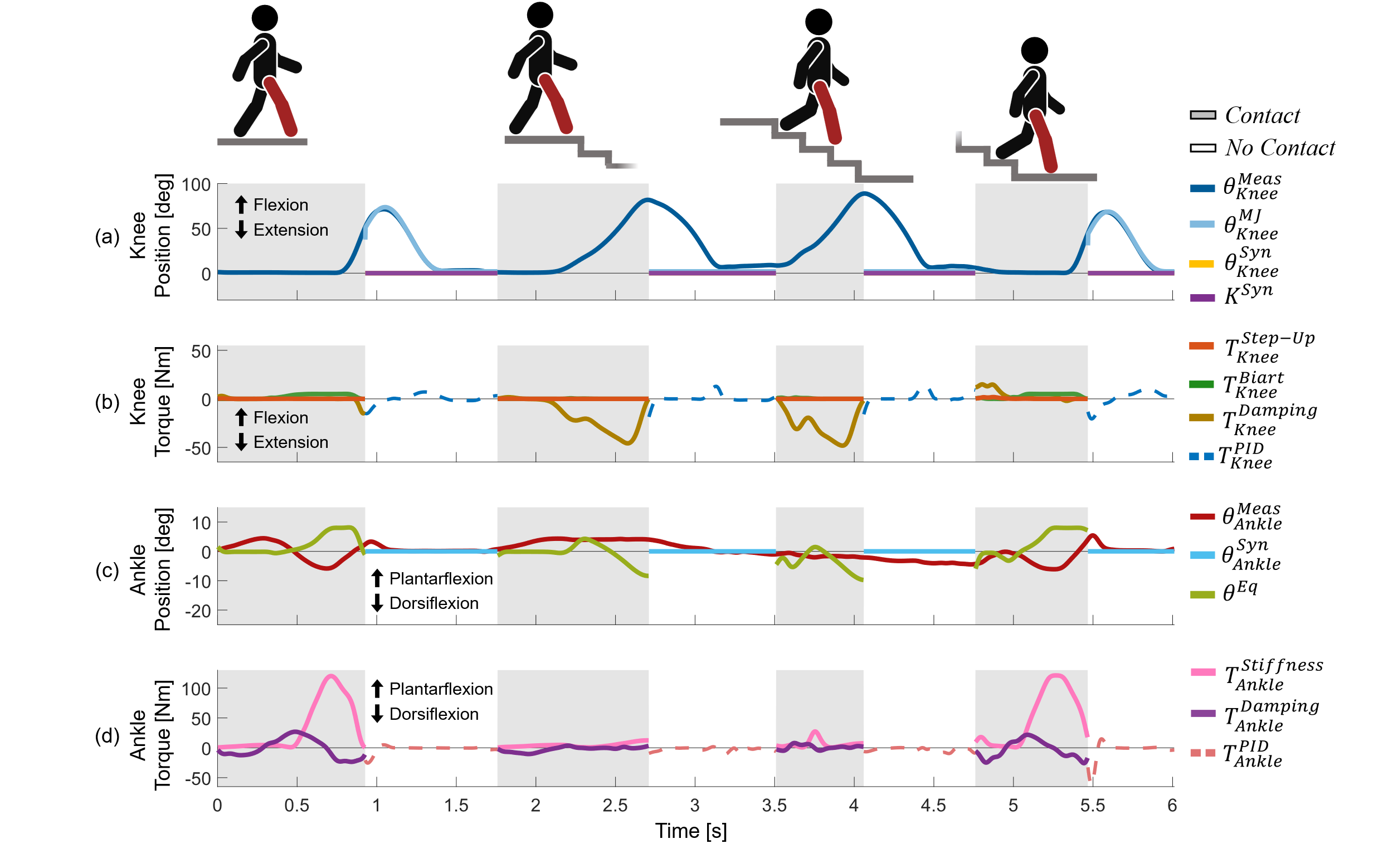


Fig. S8iii *Walk-Stair Descent-Walk PSF.* (a) Knee position, (b) knee torque, (c) ankle position, and (d) ankle torque from TF02 during a Walk-Stair Descent-Walk series of steps leading with the prosthesis.

*S9d, Walk-Stair Descent-Walk SSF*

Fig. S8iv shows joint position and torque data from TF02 during a four-step sequence in which they transition from walking to stair descent and back to walking leading with their sound limb. The first stride in the sequence is a walking stride starting from standing. During *No Contact* of the first stride, the user’s sound limb lands on the edge of the stairs and flexes as the user descends the first step. As the user’s sound limb flexes, the prosthesis extends and lands on the second step at 1.75 s. $\theta_{Knee}^{HS}$ is 1.2° and the knee flexes up to 85.4° as the user descends the step (Fig. S8iv). The kinematics are equivalent to those seen in the previous experiment when leading with the prosthesis, and the unified controller therefore commands the same control efforts (Fig. S8iv). $T_{Knee}^{Damping}$ commands up to 43.4 Nm of extension torque (Fig. S8iv). The third stride is equivalent to the second stride, and $T_{Knee}^{Damping}$ commands up to 47.3 Nm of extension torque as the knee flexes to 89.7° (Fig. S8iv). During the fourth stride, $\theta_{Knee}^{HS}$ is 4.3° and extends to 0° as the user weights the prosthesis (Fig. S8iv). These kinematics are equivalent to level ground walking, and the unified controller therefore commands the same control elements as seen in steady state walking (Fig. S3). By commanding impedance torque based on the equivalent kinematics of the user’s residual limb the unified controller enabled transitions between walking to stair descent transitions leading with the user’s sound limb.


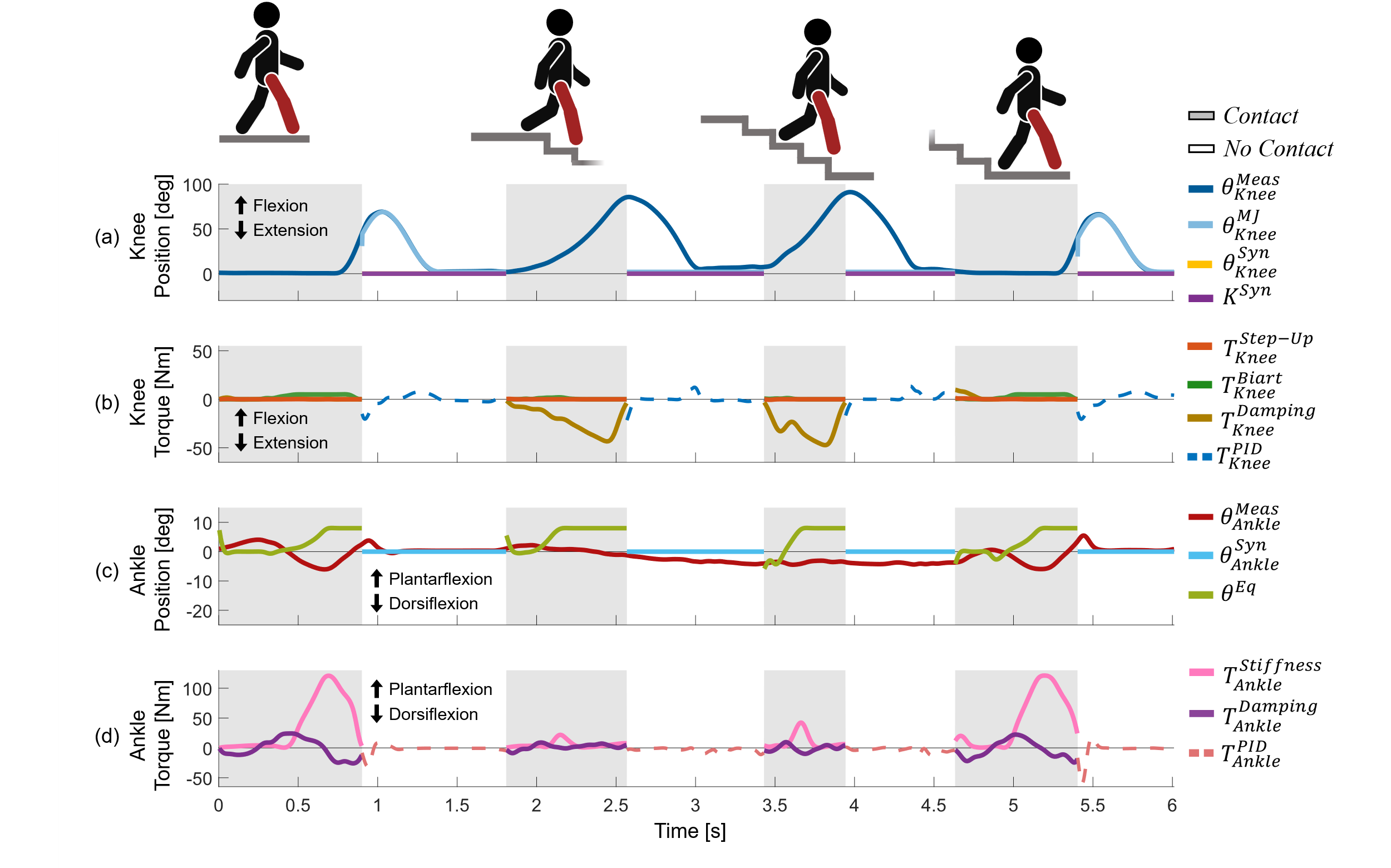


Fig. S8iv *Walk-Stair Descent-Walk SSF.* (a) Knee position, (b) knee torque, (c) ankle position, and (d) ankle torque from TF02 during a Walk-Stair Descent-Walk series of steps leading with the subject’s sound limb.
